# Enhancing the Phytoremediation of Heavy Metals by Combining Hyperaccumulator and Heavy Metal-Resistant Plant Growth-Promoting Bacteria

**DOI:** 10.3389/fpls.2022.912350

**Published:** 2022-06-02

**Authors:** Yong Zhang, Shangjun Zhao, Sijia Liu, Jing Peng, Hanchao Zhang, Qiming Zhao, Luqing Zheng, Yahua Chen, Zhenguo Shen, Xihui Xu, Chen Chen

**Affiliations:** ^1^College of Life Sciences, Nanjing Agricultural University, Nanjing, China; ^2^Quzhou Academy of Agriculture and Forestry Sciences, Quzhou Municipal Bureau of Agriculture and Rural Affairs, Quzhou, China; ^3^Jiangsu Collaborative Innovation Center for Solid Organic Waste Resource Utilization, Nanjing Agricultural University, Nanjing, China

**Keywords:** heavy metals, hyperaccumulator, PGPB-assisted phytoremediation, plant growth promoting bacteria, transposable elements

## Abstract

Heavy metals (HMs) have become a major environmental pollutant threatening ecosystems and human health. Although hyperaccumulators provide a viable alternative for the bioremediation of HMs, the potential of phytoremediation is often limited by the small biomass and slow growth rate of hyperaccumulators and HM toxicity to plants. Here, plant growth-promoting bacteria (PGPB)-assisted phytoremediation was used to enhance the phytoremediation of HM-contaminated soils. A PGPB with HM-tolerant (HMT-PGPB), *Bacillus* sp. PGP15 was isolated from the rhizosphere of a cadmium (Cd) hyperaccumulator, *Solanum nigrum*. Pot experiments demonstrated that inoculation with strain PGP15 could significantly increase the growth of *S. nigrum*. More importantly, strain PGP15 markedly improved Cd accumulation in *S. nigrum* while alleviating Cd-induced stress in *S. nigrum*. Specifically, PGP15 inoculation significantly decreased the contents of H_2_O_2_, MDA, and $O_{2}^{\cdot -}$ in *S. nigrum*, while the activities (per gram plant fresh weight) of SOD, APX, and CAT were significantly increased in the PGP15-inoculated plants compared with the control sample. These results suggested that the interactions between strain PGP15 and *S. nigrum* could overcome the limits of phytoremediation alone and highlighted the promising application potential of the PGPB-hyperaccumulator collaborative pattern in the bioremediation of HM-contaminated soils. Furthermore, the PGP15 genome was sequenced and compared with other strains to explore the mechanisms underlying plant growth promotion by HMT-PGPB. The results showed that core genes that define the fundamental metabolic capabilities of strain PGP15 might not be necessary for plant growth promotion. Meanwhile, PGP15-specific genes, including many transposable elements, played a crucial role in the adaptive evolution of HM resistance. Overall, our results improve the understanding of interactions between HMT-PGPB and plants and facilitate the application of HMT-PGPB in the phytoremediation of HM-contaminated soils.

## Introduction

In recent decades, heavy metals (HMs) have become a major environmental pollutant, as many human activities have greatly increased the release rate of HMs into the environment (Kasassi et al., 2008; Lwin et al., 2018). These accumulated HMs persist in soil for long periods and are usually non-biodegradable, which has greatly affected various ecosystems, including soil and water (Fu and Wang, 2011; Luo et al., 2011; Chen et al., 2018a; Wang et al., 2020). Although some specific HMs present in trace amounts might be essential for life as part of their key metabolic functions in biological processes, most HMs are toxic to living organisms at high concentrations. In addition, some HMs, such as cadmium (Cd), can inhibit many metabolic processes even at low concentrations, leading to deleterious effects on ecosystems and human health. For example, chronic or excessive exposure to Cd causes critical effects in humans, including lung and kidney damage (Satarug and Moore, 2004; Järup and Akesson, 2009; Johri et al., 2010). To date, Cd contamination has been widely detected in agricultural soils worldwide (Akesson et al., 2014). Although it is present at low levels in many soils, Cd is easily taken up by many crops, such as wheat, rice, and vegetables, and exposure to Cd from food can cause severe risks to human health (Akesson et al., 2014). The problems caused by Cd contamination have attracted widespread attention, and their removal from polluted environments is crucial for human health and a safe environment.

Many technologies for the remediation of HM-contaminated soils have been proposed, such as physical, chemical, and biological technologies (Ma et al., 2011; Tauqeer et al., 2016; Mishra et al., 2017; Peng et al., 2018; Verma et al., 2021). Compared to physical and chemical methods, which are costly and time-consuming and have negative impacts (Pratas et al., 2005), bioremediation-based HM hyperaccumulator plants are considered to be an inexpensive, eco-friendly, and sustainable technology for the removal of HMs from the environment. Although HM hyperaccumulator plants provide a viable alternative for the bioremediation of HMs, the potential of phytoremediation is often limited by the small biomass and slow growth rate of hyperaccumulator plants and HM toxicity (Mishra et al., 2017). Hence, promoting plant growth and alleviating HM stress in plants is essential for efficient phytoremediation.

An increasing number of studies have shown that plant growth-promoting bacteria (PGPB) can promote plant growth either directly or indirectly (Bulgarelli et al., 2013). The direct promotion of plants by PGPB usually occurs through facilitating nutrient acquisition, such as nitrogen fixation, solubilization of phosphate, and production of siderophores, or by altering phytohormone levels in plants, such as auxin, cytokinin, and ethylene (Bulgarelli et al., 2013). The indirect promotion of plant growth by PGPB generally protects plants against various pathogens or improves their resistance to environmental stresses, such as drought, salt, heavy metals, and organic contaminants (Glick, 2012). Among these PGPB, some strains have been shown to be tolerant to HMs even at high concentrations (Xu et al., 2018; He et al., 2020). Notably, in addition to altering plant cell metabolism to improve plant growth, these bacteria might also bring other benefits to host plants, such as reducing the adverse effects of HMs on plant health and allowing plants to tolerate high concentrations of HMs. In this context, HM-tolerant PGPB (HMT-PGPB), which could increase plant survival and growth in HM-contaminated soils, should have promising potential for phytoremediation (Mishra et al., 2017). Therefore, identifying and characterizing HMT-PGPB associated with hyperaccumulator plants are crucial for the enhanced phytoremediation of HM-contaminated environments. However, very few HMT-PGPB have been isolated, and data describing HMT-PGPB are limited. In addition, the interactions of HMT-PGPB with host plants in HM-contaminated soils are still largely unknown.

In this study, the bacterial strain PGP15 with Cd tolerance was isolated from the rhizosphere of a Cd hyperaccumulator plant, *Solanum nigrum*. The ability to improve plant growth and alleviate Cd stress in plants was tested. In addition, the whole genome of PGP15 was sequenced and compared with other strains to explore the molecular mechanisms behind plant growth promotion by HMT-PGPB. Our results not only improve our understanding of the interactions between HMT-PGPB and plants but will also facilitate the application of HMT-PGPB in the phytoremediation of HM-contaminated soils.

## Materials and Methods

### Bacterial Strain

The bacterial strain PGP15 used in this study was isolated from the rhizosphere soils of *S. nigrum* in a cadmium mine. The soils (10 g) were added to 90 ml of sterile water, diluted tenfold, and then spread on lysogeny broth (LB) medium. The plates were incubated at 30°C for 36 h. The obtained strains were further cultured on LB medium with Cd concentrations ranging from 0 to 1.5 mM to isolate strains with Cd tolerance. Cell growth was analyzed by measuring the optical density at 600 nm (OD600) using a Shimadzu UV-2450 (Kyoto, Japan). The relative biomass was calculated using the cell growth of cultures with different concentrations of Cd compared with the control (0 mM). The concentrations of Cd that prevented 25, 50, and 95% of cell growth were reported as the lethal dose 25 (LD25), LD50, and minimum inhibitory concentration (MIC), respectively. A Cd-resistant strain designated PGP15 was selected. To classify strain PGP15 taxonomically, its 16S rRNA gene sequence was amplified and sequenced. The taxonomic position of strain PGP15 was analyzed using the obtained 16S rRNA gene sequence by EzTaxon (Chun et al., 2007). For phylogenetic analysis, the 16S rRNA gene sequences of strain PGP15 and other related strains were aligned and manually adjusted using CLUSTALW (Thompson et al., 2002). Phylogenetic trees were constructed based on neighbor-joining and maximum likelihood methods with 1,000 replications using MEGA v7.0 (Kumar et al., 2016). The 16S rRNA gene sequence of strain PGP15 is available in GenBank under accession number ON202959.

### Plant Growth-Promoting Properties of Strain PGP15

The production of indole-3-acetic acid (IAA) by strain PGP15 was assessed in YN medium [g/L: NaCl 0.1, K_2_HPO_4_ 1.0, MgSO_4_·7H_2_O 0.5, (NH_4_)_2_SO_4_ 1.0, yeast extract 0.5, sucrose 10.0] containing 0.5 mg mL^−1^ l-tryptophan and incubated for 3 days at 30°C and 200 rpm. Then, 1.0 ml of cell suspension was thoroughly mixed with 2 ml of Salkowski’s chromogenic agent (Gordon and Weber, 1951) and allowed to stand in the dark for 30 min at 25°C. The cell suspension that was pink in color was positive for IAA production. The activity of 1-aminocyclopropane-1-carboxylate (ACC) deaminase was measured as described previously based on the production of α-ketobutyrate from ACC enzymatic hydrolysis (Belimov et al., 2005). For nitrogen fixation, strain PGP15 was incubated in a minimal medium without a nitrogen source for 7 days at 28°C according to a previous study (Navarro-Torre et al., 2016). The phosphate solubilization ability of strain PGP15 was determined by inoculating the bacterial culture in the national botanical research institute’s phosphate growth medium (NBRIP) at 28°C for 7 days, and the formation of a transparent circle around colonies was scored as positive (Navarro-Torre et al., 2016). Siderophore production was detected using blue agar medium with chrome azurol S (CAS), and the presence of an orange halo around colonies was scored as positive for the production of siderophores (Schwyn and Neilands, 1987).

The concentrations used in the organic acid assay were 0 (control), 0.0233 (LD25), 0.0538 (LD50), and 1.5848 (MIC) mM Cd. Strain PGP15 was grown in YN medium for 48 h at 30°C and 150 rpm. The cultures were then centrifuged at 6000 rpm for 10 min at 4°C to obtain the supernatant. A cationic resin (Amberlite IR-20) was used to remove Cd. The supernatants were filtered through a 0.22-μm Millipore filter and then analyzed using high-performance liquid chromatography (HPLC: LC-20AT, Shimadzu, Tokyo, Japan) with a reverse-phase C_18_ column (250 × 4.6 mm, 5 μm, Agilent, United States). The mobile phase was 20 mM monobasic potassium phosphate (pH 2.60) containing 1% methanol. The flow rate was 0.8 ml/min, and the injection volume was 20 μl. The standard curve of each organic acid was constructed, and the concentration of each organic acid was calculated by comparing the peak area with that of an organic acid standard according to the standard curve.

### Plant Growth Experiments

The growth experiment of *S. nigrum* was performed in the greenhouse of Nanjing Agricultural University. Seeds of *S. nigrum* were first sterilized in 0.5% NaClO for 10 min and then washed thoroughly with sterile deionized water (Marques et al., 2013). The seeds were germinated in sterilized vermiculite at 25°C. Following germination, seedlings were transplanted to plastic pots containing 1.5 l Hoagland nutrient solution and grown for 15 days. Uniform seedlings of *S. nigrum* at the three-leaf stage were collected and subjected to two treatments, including inoculation with strain PGP15 and the control (non-inoculated), with ten replicates per treatment. Strain PGP15 was incubated in LB at 150 rpm and 30°C for 24 h. The bacterial cells were obtained by centrifugation (8,000 rpm, 10 min) and then washed three times with sterile water followed by resuspension in sterile 0.9% NaCl solution. The cell suspension was adjusted to a final OD600 of 1.0. For inoculation, the roots of *S. nigrum* were immersed for 20 min in the bacterial solutions and then immediately transferred to plastic pots. Each pot (diameter: 15.0 cm; height: 11.5 cm) contained 1.0 kg Cd-contaminated soils, and two seedlings were transplanted into each plot. A 50-mL cell suspension was then added to the soil samples in each pot. Roots of the control treatment were immersed in sterile water. Pots were randomly placed in a greenhouse with a 13/11 h day/night photoperiod at 25–30°C. Thirty days after inoculation, plants were carefully harvested from the pots for further analysis.

### Biomass and Cd Concentration in Plants

To remove dust and soil from the plant surface, the samples were first rinsed with tap water and then washed with distilled water. In addition, to remove HMs adhering to root surfaces, the root samples were immersed in 20 mM L^−1^ EDTA-Na_2_ solution for 30 min. The dry weights of shoots and roots were measured after 48 h of drying at 80°C. The dried shoots and roots were also used for the analysis of Cd concentrations in plant tissues. After grinding thoroughly, 0.2 g plant samples were digested with a 3 ml acid mixture composed of HNO_3_ and HClO_4_ (87:13, *v*/*v*). Concentrated HNO_3_ (2.5%) was added to the digested samples to a final volume of 10 ml. The Cd concentration was measured using an Agilent 7,800 ICP mass spectrometer (Agilent, Santa Clara, CA, United States).

### Determination of Lipid Peroxidation, Proline, GSH, $O_{2}^{\cdot -}$ , and H_2_O_2_

Lipid peroxidation was measured by assessing the reaction of malondialdehyde (MDA) to thiobarbituric acid (TBA) using the method described by Chen et al. (2017) with minor modifications. A 0.5 g fresh sample was homogenized in 5 ml of 10% (w/v) trichloroacetic acid (TCA) and centrifuged at 20,000 rpm for 25 min. We then added 2 ml of supernatant to 2 ml of 0.67% (w/v) TBA containing 10% TCA. The mixture was heated at 95°C for 30 min and then cooled rapidly in an ice bath. Following centrifugation at 12,000 rpm for 10 min, the absorbance of the supernatant was recorded at 450, 532, and 600 nm. The concentration of MDA was calculated as follows: MDA concentration (μmol/g FW) = [6.45 × (A532 − A600) − 0.56 × A450] × v/w, where *v* represents the volume of the extraction solution.

Proline (Pro) was measured as described by Bates et al. (1973) with minor modifications. Approximately 0.5 g of fresh sample was homogenized in 10 ml of 3% (w/v) aqueous sulfosalicylic acid. We then added 2 ml of filtrate to 2 ml of glacial acetic acid and 2 ml of acid ninhydrin. The mixture was first heated at 100°C for 10 min and then cooled rapidly in an ice bath. The reaction mixture was extracted with 4 ml toluene and mixed vigorously for 30 s. Following centrifugation at 3,000 rpm for 5 min, the absorbance of the supernatant was recorded at 520 nm. Pro was determined from a standard curve and expressed as μg/g fresh weight (FW).

Reduced glutathione (GSH) was determined with commercial GSH assay kits (Beijing Solarbio Science & Technology Co., Ltd., Beijing, China). GSH was measured by assessing the absorbance of the reaction mixture at 412 nm.

The content of $O_{2}^{\cdot -}$ was determined using the method described by Jiang and Zhang (2001). The production rate of $O_{2}^{\cdot -}$ was measured by monitoring the absorbance at 530 nm during nitrite formation from hydroxylamine hydrochloride in the presence of $O_{2}^{\cdot -}$ . The content of H_2_O_2_ was determined according to Velikova et al. (2000). The H_2_O_2_ content was measured by assessing the absorbance of the titanium peroxide complex at 390 nm. Absorbance values were calibrated to a standard curve generated using a known concentration of H_2_O_2_.

### Antioxidant Enzyme Activities

The enzymatic antioxidant activity of *S. nigrum* was investigated by carrying out superoxide dismutase (SOD), peroxidase (POD), ascorbate peroxidase (APX), and catalase (CAT) activity assays. The method of enzyme extraction followed Wang et al. (2019). Fresh leaf segments (0.5 g) were homogenized in 5 ml precooled 100 mM potassium phosphate buffer (pH 7.0) containing 1 mM ethylenediaminetetraacetic acid disodium salt (EDTA-Na_2_) and 1% polyvinylpyrrolidone (PVP). The homogenate was centrifuged at 12,000 rpm for 20 min at 4°C, and the supernatant was used for the enzyme assays below.

SOD activity was assayed by monitoring the photochemical reduction inhibition of nitroblue tetrazolium (NBT) following the method of Giannopolitis and Ries (1977). One unit of SOD activity was defined as the amount of enzyme required to inhibit NBT reduction by 50% as monitored at 560 nm. POD activity was measured according to the method of Kenten and Mann (1954) and modified by Chen et al. (2017). The growing absorbance at 470 nm was monitored for 1 min as guaiacol was oxidized, and the extinction coefficient was 26.6 mM^−1^ cm^−1^. APX activity was measured as described by Nakano and Asada (1981). The oxidation of ascorbate was followed by a decrease in the absorbance at 290 nm for 3 min. CAT activity was determined by monitoring the reduction of H_2_O_2_ at 240 nm within 3 min (Aebi, 1984).

### Measurements of Pigment and Gas Exchange

Photosynthetic pigment content was determined using the method described by Knudson et al. (1977). Photosynthetic pigments were extracted by soaking a 0.1 g leaf sample in 10 ml of 95% ethanol and measuring the absorption of the extracts at 470, 649, and 665 nm using spectrophotometry (UV-2450, Shimadzu, Kyoto, Japan). Chlorophyll a, chlorophyll b, and total carotenoid contents were calculated using equations from Lichtenthaler and Wellburn (1983), and photosynthetic gas exchange parameters were measured according to Chen et al. (2017). Photosynthetic gas exchange parameters, including net photosynthetic rate (Pn), intercellular CO_2_ concentration (Ci), stomatal conductance (Gs), and transpiration rate (Tr), were measured between 08:30 and 11:30 using a Li-6,400 portable photosynthesis system (LI-COR Biosciences, Lincoln, NE, United States) equipped with an LED light source.

### Genome Sequencing and Annotation

Whole-genome sequencing of strain PGP15 was performed at Wuhan Benagen Tech Solutions Company Limited (Wuhan, China) by a combination of second-generation Illumina and third-generation Nanopore technologies (Jain et al., 2018). Low-quality reads were filtered using GUPPY v5.0.16^1^ and SOAPnuke v2.1.2 (Chen et al., 2018b). A total of 1,000,025,449 bp and 1,745,824,200 bp of clean data were obtained by Nanopore and Illumina sequencing, respectively. Unicycler v0.4.9 (Wick et al., 2017) was used to assemble the filtered reads, which were then assembled into three contigs (a chromosome and two plasmids) without gaps. The complete genome sequence of PGP15 was annotated using Prokka v1.12 (Seemann, 2014). The gene functions were predicted using eight databases, including UniProt, Pfam, Refseq, Non-redundant (NR), Tigrfam, Gene Ontology (GO), Kyoto Encyclopedia of Genes, and Genomes (KEGG), and Clusters of Orthologous Groups (COG). The whole-genome sequences of strain PGP15 are available in GenBank under accession numbers CP095874–CP095876.

### Comparative Genomic Analysis

For the comparative genome analysis of strain PGP15 and closely related species, the genome sequences of three *Bacillus* strains, including *Bacillus wiedmannii* SR52, *Bacillus mycoides* BPN36/3, and *Bacillus cereus* BC33, were downloaded from NCBI. The average nucleotide identity (ANI) value was calculated by using JSpecies v3.9.1 with Blast (ANIb) and MUMmer (ANIm) under the default parameters (Richter et al., 2016). Pairwise genomic sequence alignment was performed using MUMmer v4.0.0beta2 (Kurtz et al., 2004) to identify genome-wide rearrangements and inversions. To identify local collinear blocks (LCBs) between the *PGP15* genome and other query genomes, multiple comparisons of the genomes were performed using MCScanx (Wang et al., 2012), with at least five homologous genes and fewer than ten gaps required to call an LCB. To identify orthologous genes among the four query species, proteins were clustered using OrthoMCL v2.0.9 (Li et al., 2003). Ortholog, coortholog, and inparalog pairs were identified and then used to construct an OrthoMCL graph for clustering with the Markov cluster (MCL) algorithm (Enright et al., 2002).

### Statistical Analyses

Microsoft Excel 2010 (Microsoft Corp., Albuquerque, NM, United States) and SPSS v22.0 (IBM, Endicott, NY, United States) were used for data analyses. Significant differences were determined using Duncan’s test at *p* < 0.05. Graphical work was performed in GraphPad Prism 6.0 (GraphPad Inc., San Diego, CA, United States).

## Results

### Identification of Strain PGP15 and Characterization of Its Cd Tolerance

**Figure 1:**
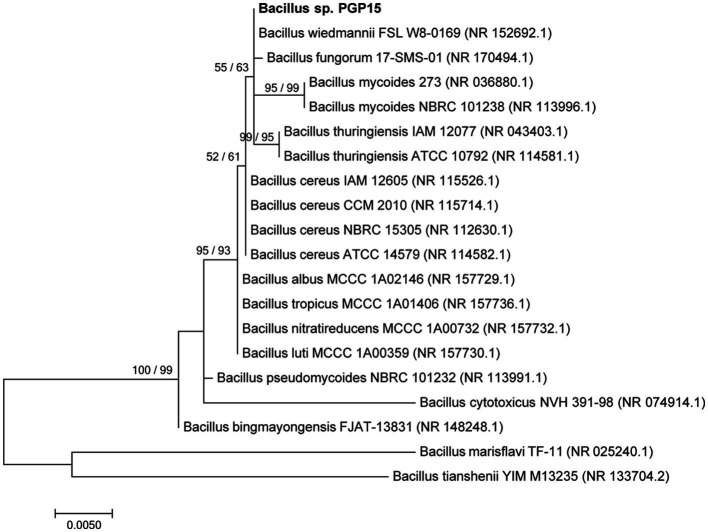
Phylogenetic relationships between strain PGP15 and related species showing the position of strain PGP5 within the genus *Bacillus*. The maximum likelihood (ML) phylogenetic tree based on 16S rRNA gene sequences is shown. The ML and neighbor-joining bootstrap values based on 1,000 replications are sequentially indicated above the branches. Bar, 0.005 substitutions per nucleotide position.

Strain PGP15 was isolated from Cd-contaminated soils in the rhizosphere of *S. nigrum*. Based on comparisons of the 16S rRNA gene sequences, strain PGP15 showed 99.11% similarity with strain *B. wiedmannii* FSL W8-0169. Meanwhile, the phylogenetic analysis based on 16S rRNA gene sequences showed that strain PGP15 was clustered in a group consisting of *B. wiedmannii*, *B. fungorum*, *B. mycoides*, and *B. thuringiensis* (Figure 1). These results indicated that strain PGP15 could be assigned to the genus *Bacillus*, belonging to the family Bacillaceae.

**Figure 2:**
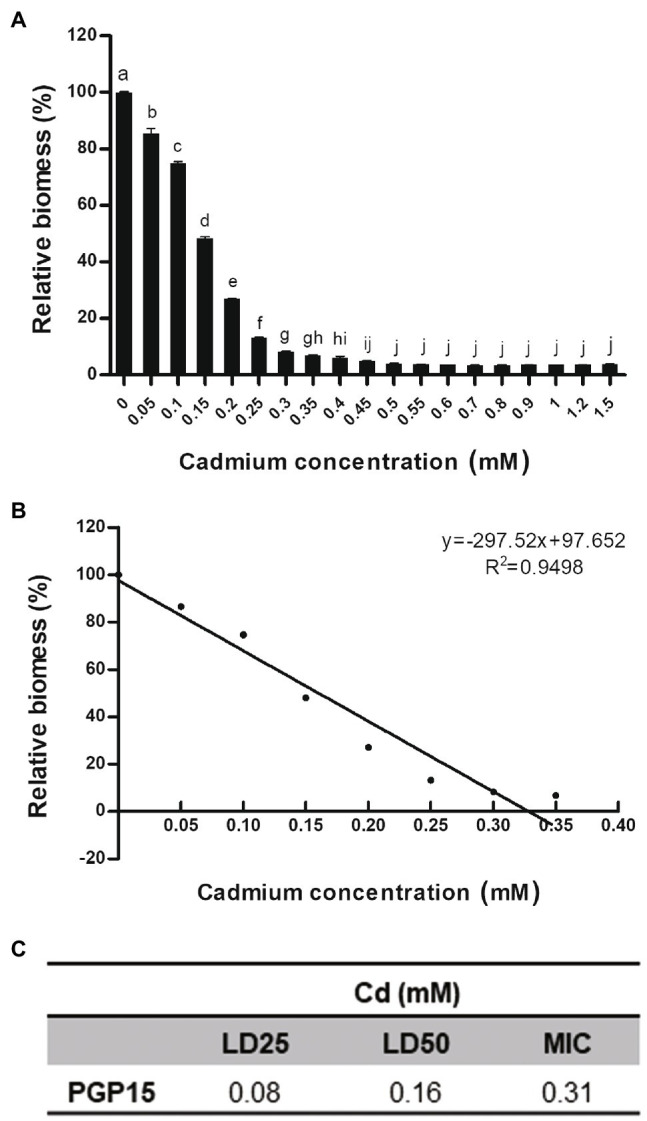
The Cd resistance profile for strain PGP15. **(A)** Relative biomass of strain PGP15 during cadmium treatments. **(B)** Linear relationship between the relative biomass of strain PGP15 and the cadmium concentrations in the medium. The growth of strain PGP15 was studied under a gradient of cadmium concentrations. The relative biomass was calculated by comparing the biomass (OD600) of different treatments with that of the control. Error bars are standard errors of the means of three replicates. **(C)** The LD25, LD50, and MIC of strain PGP15 when exposed to Cd. LD, lethal dose; MIC, minimum inhibitory concentration.

To test its Cd tolerance, strain PGP15 was assayed for its ability to grow in the presence of Cd at different concentrations (Figure 2). The growth of strain PGP15 was gradually inhibited with increasing concentrations of Cd. The inhibitory effects of Cd on strain growth were measured, and the LD25, LD50, and MIC were 0.08, 0.16, and 0.31 mM, respectively.

### Plant Growth Promotion by Strain PGP15

**Figure 3:**
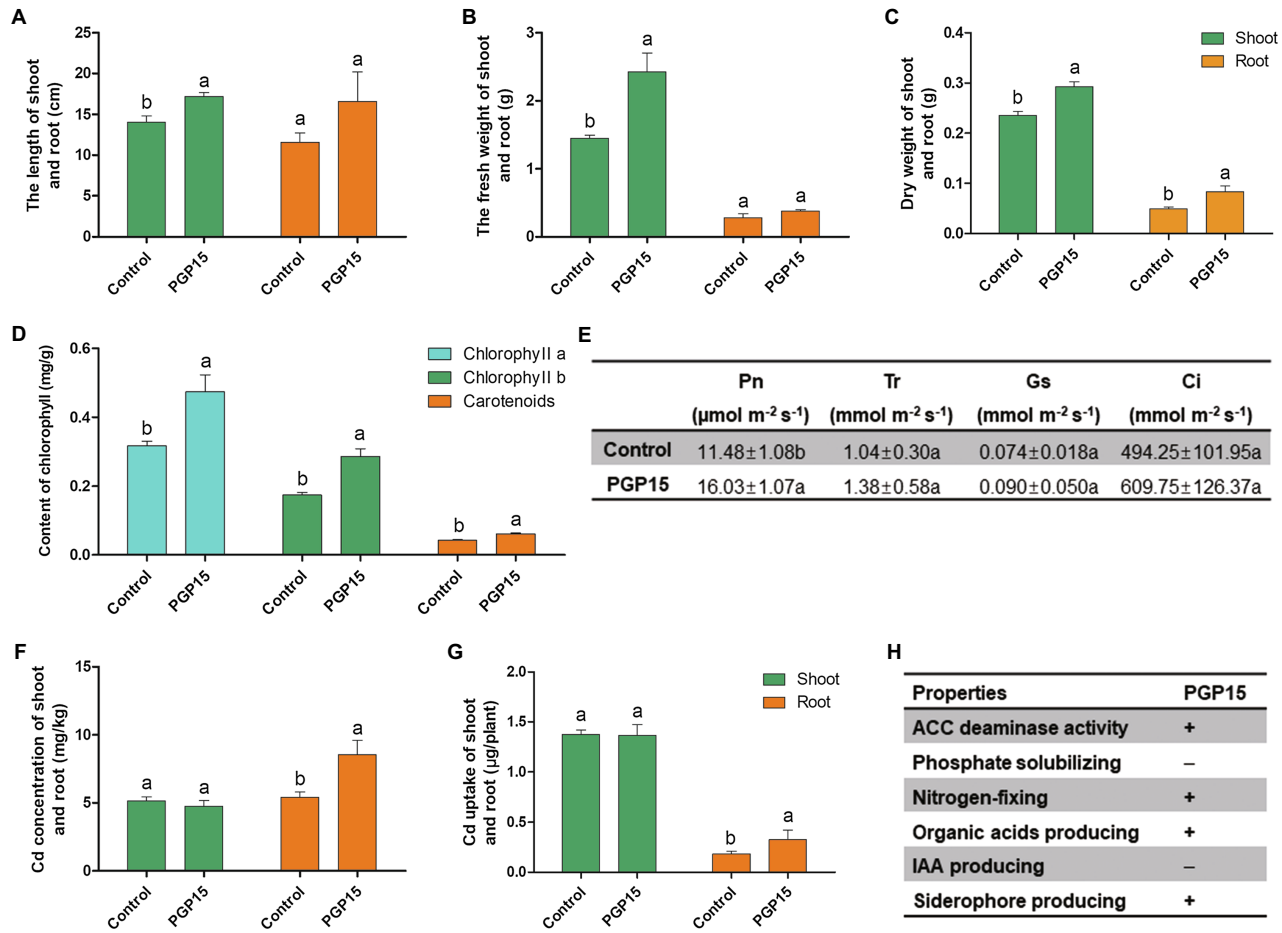
Promotion of growth, Cd accumulation and alleviation of Cd-induced stress in *Solanum nigrum* by strain PGP15. The lengths **(A)** fresh weights **(B)** and dry weights **(C)** of shoots and roots are shown. **(D)** Chlorophyll a, chlorophyll b and carotenoid contents. **(E)** The effects of PGP15 inoculation on photosynthesis rate (Pn), transpiration rate (Tr), stomatal conductance (Gs), and intercellular CO_2_ concentration (Ci). **(F)** The Cd concentrations in shoots and roots. **(G)** Total Cd accumulation in shoots and roots. **(H)** Basic growth-promoting properties of strain PGP15. The activities detected in strain PGP15 are indicated with a plus sign. IAA, indole-3-acetic acid; ACC, 1-aminocyclopropane-1-carboxylate.

To evaluate the ability of strain PGP15 to promote plant growth under Cd stress, strain PGP15 was inoculated into Cd-polluted rhizosphere soils of *S. nigrum*. Compared with control plants without inoculation, the length, fresh weight, and dry weight of shoots from inoculated plants increased significantly by 29.19, 67.36, and 24.28%, respectively (Figures 3A–C). No significant increase in root length or fresh weight was detected in the inoculated vs. control plants. However, the dry weight of roots from inoculated plants significantly increased by 68.79% compared with control plants. In addition, compared with control plants, the chlorophyll a, chlorophyll b, and carotenoid contents in inoculated plants were significantly increased by 49.53, 64.31, and 45.04%, respectively (Figure 3D). Consistently, the photosynthesis rate, transpiration rate, stomatal conductance, and intercellular CO_2_ concentration in the PGP15-inoculated *S. nigrum* increased by 39.63, 32.69, 21.62, and 23.37%, respectively (Figure 3E). These results showed that PGP15 significantly improved plant growth under Cd stress.

Multiple plant growth-promoting properties of PGPB were detected in strain PGP15 (Figure 3H). Strain PGP15 was capable of producing siderophores and organic acids and possessed nitrogen fixation capability but was not capable of producing IAA or solubilizing phosphate. In addition, the strain also had ACC deaminase activity.

The ability of strain PGP15 to produce organic acids in the extracellular space under different Cd concentrations was further analyzed (Table 1). Without Cd stress, low levels of organic acids, including oxalic acid, formic acid, acetic acid, and citric acid, were produced by strain PGP15 (Table 1). However, when exposed to Cd at LD25 or LD50 levels, the production levels of these acids were significantly increased. The increased production of organic acids could participate in metal binding and their function as detoxification agents, as organic acids could potentially reduce the HM concentration in the cytoplasm (Jones, 1998; Chen et al., 2016). At the MIC level of Cd, decreased production of organic acids was detected. One possible explanation was that the survival of strain PGP15 was suppressed by the high concentration of Cd.

**Table 1:** Organic acid production by strain PGP15 under different cadmium treatments.

	Oxalic acid (mg/L)	Tartaric acid (mg/L)	Formic acid (mg/L)	Malic acid (mg/L)	Acetic acid (mg/L)	Citric acid (mg/L)	Succinic acid (mg/L)
0	2.96 ± 0.18c	0	20.63 ± 0.61c	0	1.40 ± 0.27c	10.35 ± 0.95b	0
LD25	3.38 ± 0.04b	0	680.61 ± 104.46a	0	0	32.84 ± 4.62a	0
LD50	3.62 ± 0.02a	0	285.98 ± 10.77b	0	17.76 ± 0.11a	8.41 ± 1.15b	0
MIC	2.79 ± 0.10d	0	29.10 ± 1.62c	0	10.34 ± 1.23b	0	0

*The LD25, LD50, and MIC of strain PGP15 exposed to Cd are the same as those in Figure 2. LD, lethal dose; MIC, minimum inhibitory concentration. Different letters indicate statistically significant differences (*p* < 0.05)*.

### Inoculation With Strain PGP15 Increased Cd Accumulation in Plants

The Cd concentrations in both the shoots and roots of *S. nigrum* were further measured to analyze the effect of Cd accumulation in plants by inoculation with strain PGP15. Although no significant difference in Cd concentration in shoots was detected, inoculation with strain PGP15 significantly increased the Cd concentration in roots (Figure 3F). Similar results were found for the total amount of Cd accumulation, that is, the Cd accumulation in inoculated roots was significantly higher than that in roots without inoculation (Figure 3G). Combined with the improved biomass of inoculated plants, these results showed the possibility of enhancing phytoremediation by combining PGPB and a hyperaccumulator and the promising potential of strain PGP15 for application in phytoremediation.

### Alleviation of Cd-Induced Stress in Plants by Strain PGP15

To assess the oxidative stress in *S. nigrum* under Cd stress, the contents of H_2_O_2_, MDA, and $O_{2}^{\cdot -}$ in *S. nigrum* were detected. To minimize the damaging effects of HM-induced reactive oxygen species (ROS), many plants can produce various enzymes (e.g., SOD, APX, CAT, and POD) and non-enzymatic antioxidants (e.g., GSH and Pro) to scavenge ROS. Therefore, these enzymatic and non-enzymatic antioxidants of *S. nigrum* were analyzed to study the effects of PGP15 inoculation on the ROS-scavenging system (Table 2). The results showed that PGP15 inoculation significantly decreased the contents of H_2_O_2_, MDA, and $O_{2}^{\cdot -}$ in *S. nigrum*. The results showed that Cd triggered oxidative stress in *S. nigrum*, and the Cd-induced oxidative stress in *S. nigrum* was at least partly alleviated by PGP15 inoculation. Meanwhile, the activities of SOD, APX, and CAT were significantly increased in the PGP15-inoculated plants compared with the control samples. No significant increase in POD, GSH, or Pro was detected in PGP15-inoculated plants. Together, these results showed that inoculation with strain PGP15 could significantly alleviate Cd stress in *S. nigrum*.

**Table 2:** The effects of Cd treatment on H_2_O_2_, malondialdehyde (MDA), and $O_{2}^{\cdot -}$ concentrations in shoots and roots and the enzymatic (SOD, APX, CAT, and POD) and non-enzymatic antioxidants (GSH and Pro) in shoots of *S. nigrum*.

	Shoot		Root	
	Control	PGP15	Control	PGP15
H_2_O_2_ (μmol g^−1^ FW)	64.87 ± 12.20a	45.60 ± 7.86a	20.70 ± 3.39a	15.42 ± 0.94b
MDA (μmol kg^−1^ FW)	16.60 ± 1.18a	14.13 ± 1.56b	45.26 ± 4.79a	33.67 ± 3.27b
$O_{2}^{\cdot -}$ (μmol g^−1^ FW)	6.40 ± 0.68a	5.03 ± 0.49 b	5.08 ± 0.40a	3.45 ± 0.09b
SOD (U g^−1^ FW)	97.97 ± 1.62b	111.77 ± 4.771a	–	–
APX (μmol min^−1^ g^−1^ FW)	1.14 ± 0.06b	1.54 ± 0.06a	–	–
CAT (μmol min^−1^ g^−1^ FW)	60.82 ± 2.33b	91.85 ± 7.22a	–	–
POD (ΔOD_470_·min^−1^ g^−1^ FW)	0.27 ± 0.07a	0.42 ± 0.17a	–	–
GSH (μg g^−1^ FW)	170.93 ± 18.01a	223.00 ± 33.45a	–	–
Pro (μg g^−1^ FW)	23.64 ± 2.31a	14.33 ± 1.74b	–	–

*H_2_O_2_, hydrogen peroxide, MDA, malondialdehyde;* $O_{2}^{\cdot -}$ , *superoxide radical; SOD, superoxide dismutase; POD, peroxidase; CAT, catalase; APX, ascorbate peroxidase; GSH, glutathione; Pro, proline; FW, fresh weight. Different letters indicate that values are significant different at *p* < 0.05*.

### Genome Assembly and Annotation of Strain PGP15

**Figure 4:**
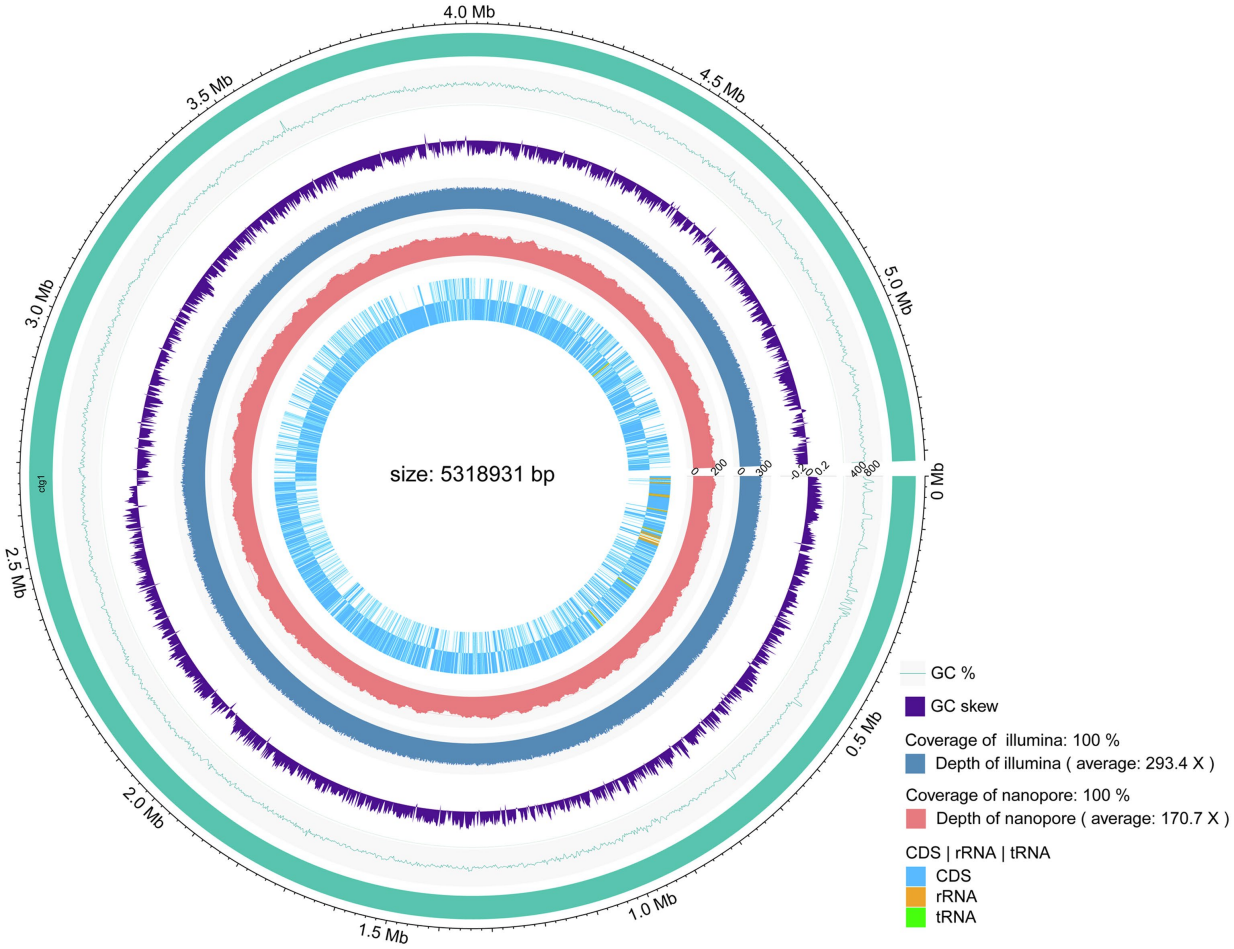
Circular representation of the PGP15 chromosome. The peripheral circle represents the genome with sizes marked in Mb. The second and third circles show the GC content and GC skew, respectively. The fourth and fifth circles indicate the coverage levels of Illumina and Nanopore sequencing, respectively. The inner two circles represent the distribution of predicted CDSs, tRNAs, and rRNAs.

**Figure 5:**
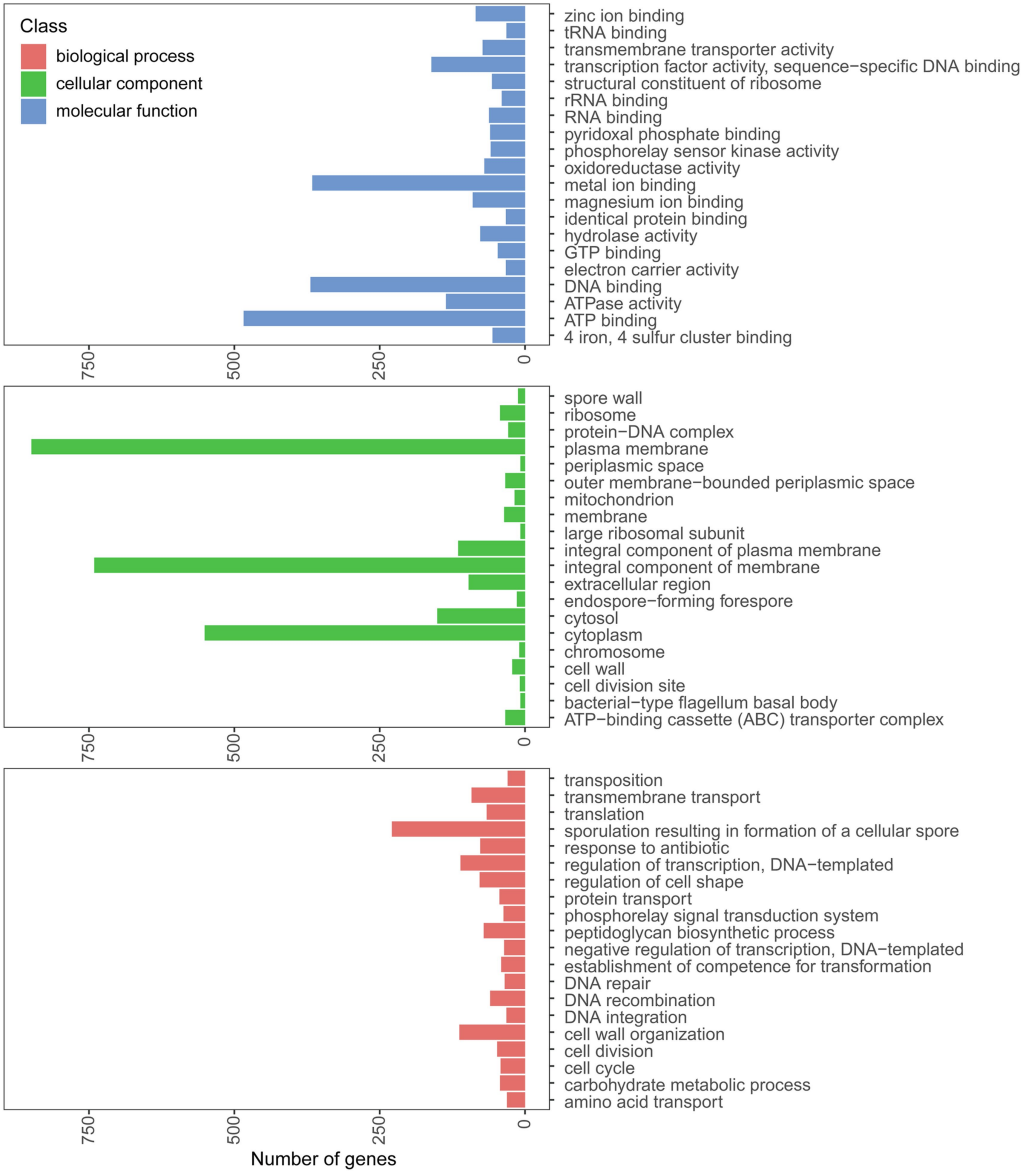
Annotation of genes in the PGP15 genome. The GO categories belonging to biological process, cellular component, and molecular function are shown.

The genome of strain PGP15 contained one circular chromosome and two circular plasmids (Figure 4; Table 3). The length of the chromosome was 5,318,931 bp, with an average G + C content of 35.5%. The two plasmids contained 525,898 and 9,385 bp, respectively. In total, the genome of strain PGP15 encoded 6,124 genes. In addition, 42 rRNA and 106 tRNA genes were detected in the genome. In total, 5,834 genes (95.26% of total genes) were functionally annotated according to the results of comparisons with public databases (Table 3). Among them, 5,788 (94.51%), 5,020 (81.97%), 4,266 (69.66%), 3,558 (58.10%), and 3,268 (53.36%) genes were compared in the RefSeq, Pfam, NR, UniProt, and GO databases, respectively (Supplementary Table S1). However, 633 (10.33%) genes were annotated with “hypothetical protein.” Specifically, GO annotations were used to provide functional insight into the predicted genes (Figure 5). According to the three primary GO categories (biological process, cellular component, and molecular function), genes associated with ATP binding (464), DNA binding (369), and metal ion binding (366) were abundant within the molecular function category; genes associated with the plasma membrane (849), integral component of the membrane (741), and cytoplasm (551) were abundant within the cellular component category; and genes associated with sporulation resulting in the formation of a cellular spore (229), cell wall organization (113), and regulation of transcription (111) were abundant within the biological process category. An additional 161, 73, and 70 genes were found to be involved in transcription factor activity, transmembrane transporter activity, and oxidoreductase activity, respectively.

**Table 3:** Genome features of strain PGP15.

	Chromosome	Plasmid 1	Plasmid 2
Genome size (bp)	5,318,931	525,898	9,385
GC content (%)	35.5	33.3	33.3
Total numbers of genes	5,681	435	8
Contig numbers	1	1	1
rRNA genes	42	0	0
tRNA genes	105	1	0

The genome of strain PGP15 was found to contain many candidate genes related to plant growth promotion (Table 4). For example, genes involved in nitrogen fixation, acetolactate synthase, and siderophore biosynthesis were detected. Meanwhile, genes related to auxin synthesis, such as ipdC, or genes functional in phosphate solubilization, such as pqqEFG, were not found in the PGP15 genome. These results were consistent with the plant growth-promoting properties of strain PGP15 (Figure 3H). In addition, the PGP15 genome also contained genes involved in cobalamin and petrobactin biosynthesis (Table 4).

**Table 4:** Predicted genes associated with plant growth promotion in PGP15 and the three other genomes.

Gene ID	Name	OG ID	Bac	Bam	Baw	Function
g_05163	*nifU*	OG0969	WP_000431159.1	WP_000431159.1	WP_000431158.1	Nitrogen fixation
g_01763	*cobW*	OG4974	WP_149287505.1	–	–	Cobalamin biosynthesis
g_01820	*cbiN*	OG4588	–	WP_098168118.1	WP_000831707.1	Cobalamin biosynthesis
g_05560	*speB*	OG2890	WP_001209831.1	WP_002016350.1	WP_001209823.1	Agmatinase
g_01435	*ilvB*	OG0254	WP_000095869.1	WP_215552697.1	WP_000095842.1	Acetolactate synthase
g_01436	*ilvH*	OG1988	WP_000822952.1	WP_000822948.1	WP_000822948.1	Acetolactate synthase
g_01845	*ilvB*	OG4315	WP_002182646.1	–	WP_000434918.1	Acetolactate synthase
g_01846	*ilvN*	OG4029	WP_000019917.1	–	WP_002108560.1	Acetolactate synthase
g_00908	*alsS*	OG3464	WP_074554189.1	WP_060750040.1	WP_064459733.1	Acetolactate synthase
g_05723	*ilvB*	–	–	–	–	Acetolactate synthase
g_01983	*rhbC*	OG2796	WP_001163345.1	WP_078184741.1	WP_151035405.1	Siderophore biosynthesis
g_01984	*rhbC*	OG3954	WP_211478018.1	WP_215552499.1	WP_001261840.1	Siderophore biosynthesis
g_01987	*asbE*	OG0474	WP_000200705.1	WP_063217767.1	WP_000200724.1	Petrobactin biosynthesis
g_01986	*asbD*	OG2979	WP_001250560.1	WP_063217768.1	WP_000818071.1	Petrobactin biosynthesis

*OG, ortholog groups identified by OrthoMCL; Bac, *B. cereus* BC33; Bam, *B. mycoides* BPN36/3; Baw, *B. wiedmannii* SR52*.

### Comparative Genomics of *Bacillus*

**Figure 6:**
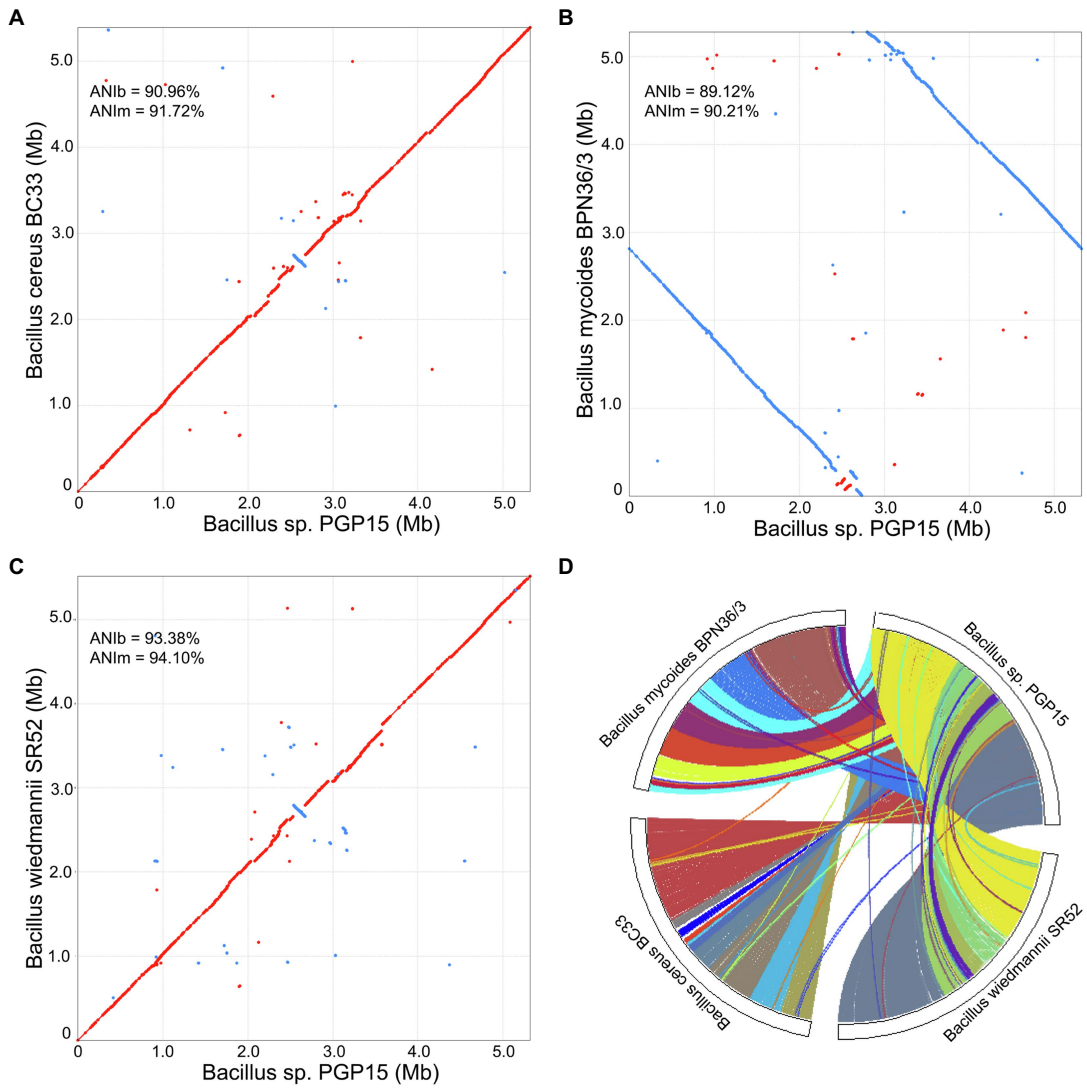
Collinearity analysis of chromosomes of strains PGP15, *B. cereus* BC33, *B. wiedmannii* SR52, and *B. mycoides* BPN36/3. **(A–C)** Complete genome alignments of strain PGP15 against *B. cereus* BC33 **(A)**, *B. mycoides* BPN36/3 **(B)**, and *B. wiedmannii* SR52 **(C)** using Mummer software. Red and blue dots represent forward and reverse matches, respectively. The average nucleotide identity (ANI) was analyzed by pairwise comparison based on Blast (ANIb) and MUMmer (ANIm). **(D)** Genome synteny of the four strains based on MCScanX analysis, showing the conserved genomic regions shared among them. Each line represents a syntenic block of five or more gene pairs.

To investigate the mechanisms underlying plant growth promotion by strain PGP15, comparative genomic analysis was performed among strain PGP5 and three other closely related strains, *B. wiedmannii* SR52, *B. mycoides* BPN36/3, and *B. cereus* BC33. The genomic DNA G + C content of strain PGP15 (35.5%) was similar to the contents of *B. wiedmannii* SR52 (35.4%), *B. mycoides* BPN36/3 (35.6%), and *B. cereus* BC33 (35.4%). The ANI was analyzed by pairwise comparison with both ANIb and ANIm. The ANIb values between strain PGP5 and *B. wiedmannii* SR52, *B. cereus* BC33, or *B. mycoides* BPN36/3 were 93.38, 90.96, and 89.12%, respectively (Figure 6). Strain PGP15 shared 94.10, 91.72, and 90.21% identity of ANIm with *B. wiedmannii* SR52, *B. cereus* BC33, and *B. mycoides* BPN36/3, respectively (Figure 6). The ANI values were below the suggested cutoff value of 95% to delineate bacterial species. However, these results showed high similarity between the genomes of strain PGP15 and the other three tested strains, especially for *B. wiedmannii* SR52.

A collinearity analysis was performed to further compare the similarities and differences between the genome of strain PGP15 and the other three strains (Figure 6). Generally, the PGP15 genome displayed high synteny to those of the three other strains. However, variations between the genomes were detected, such as insertions and deletions (Figure 6). Moreover, a major inversion was detected between PGP15 and the other three strains (Figures 6A–C). A total of 28 LCBs were observed in the synteny comparison of PGP15 vs. *B. wiedmannii* SR52, containing 4,041 genes of PGP15 (Figure 6D). Similarly, 17 and 24 LCBs were detected in the comparison of PGP15 vs. *B. cereus* BC33 and *B. mycoides* BPN36/3, which contained 4,357 and 4,132 genes of PGP15, respectively (Figure 6D). Together, the PGP15 genome showed the highest synteny with *B. wiedmannii* SR52, indicating that their genomes were closely related, which was consistent with the results of ANI and the phylogenetic tree.

**Figure 7:**
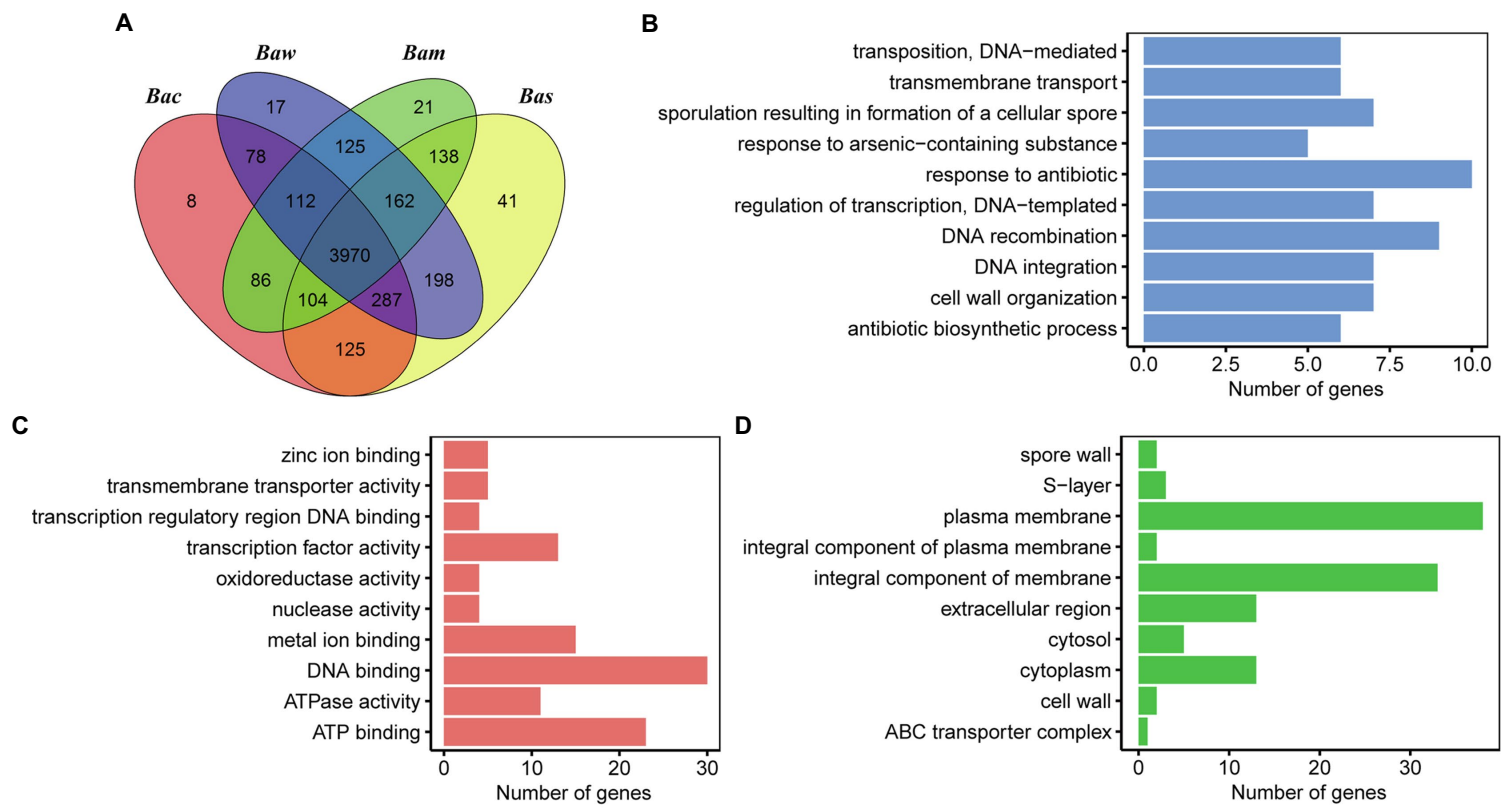
Comparison of orthologous genes in genomes of strains PGP15, *B. cereus* BC33, *B. wiedmannii* SR52, and *B. mycoides* BPN36/3. **(A)** Venn diagram representing the numbers of shared and unique ortholog groups among the four genomes. The ortholog groups identified by OrthoMCL software are shown. Bac, *B. cereus* BC33; Bam, *B. mycoides* BPN36/3; Bas, *Bacillus* sp. PGP15; Baw, *B. wiedmannii* SR52. **(B–D)** GO annotation of unique genes (i.e., genes are not included in any ortholog groups) in the PGP15 genome. The three main GO categories, including biological process **(B)**, molecular function **(C)**, and cellular component **(D)**, are shown.

To explore the orthologs and unique genes among PGP15 and the other three genomes, an MCL analysis was performed. A total of 5,471 orthologous groups (OGs, containing 19,962 genes in total) were clustered between strain PGP15 and the other three genomes (Figure 7). Notably, most OGs (3,970) were shared by all four genomes, forming core groups that were conserved among the four strains (Figure 7). A total of 5,231 (89.7%) genes of PGP15 clustered into 5,025 OGs. Similarly, most of the genes in the other three genomes were included in the OGs: 5031 (91.3%, 4,948 OGs), 4,839 (90.5%, 4,769 OGs) and 4,861 (89.6%, 4,717 OGs) genes for *B. wiedmannii* SR52, *B. cereus* BC33, and *B. mycoides* BPN36/3 were clustered into OGs, respectively. These results showed high similarity among the four genomes, which was consistent with the synteny results.

Notably, a total of 41 OGs (110 genes) were specific to strain PGP15, which was much more than those of the other three genomes (8, 17, and 21 OGs for *B. cereus* BC33, *B. wiedmannii* SR52, and *B. mycoides* BPN36/3, respectively, Figure 7). Interestingly, most of the PGP15-specific OGs, especially the large OGs with more than 3 genes, were transposon-related (e.g., insertion sequence, IS; Supplementary Table S2). The results suggested that these transposable elements (TEs) might have an important influence on the dynamics of the evolution of the PGP15 genome. Another 42 genes without annotation or that were annotated as hypothetical proteins were identified.

The unique genes for PGP15 that had no orthologs in MCL analysis were further explored (Supplementary Table S3). A total of 703 PGP15 unique genes were identified, including 450 genes from chromosomes and 253 genes from plasmids. Most of these genes were without annotation based on database comparison (Figure 7A; Supplementary Table S3). For example, 161 and 176 genes were annotated based on the GO and UniProt databases, respectively (Figures 7B–D; Supplementary Table S3). For the category of biological process, genes involved in DNA recombination, DNA integration, transposition, and DNA-mediated were abundant (Figure 7B), suggesting that these genes were involved in TEs. Five genes were associated with the response to arsenic-containing substances, indicating that the strain might have the ability to resist arsenic. Metal ion binding was abundant within molecular function (Figure 7C), and plasma membrane and integral component of membrane were abundant within cellular component (Figure 7D), suggesting these genes might be functional in metal transportation across the plasma membrane. Taken together, the GP15-specific OGs and unique genes were important for the evolution of the PGP15 genome, which could also help to improve the resistance to heavy metals.

## Discussion

Plant-beneficial rhizobacteria have been important for sustainable agriculture due to their promising potential for application in agriculture (Parnell et al., 2016; Cordovez et al., 2019; Chen et al., 2022). The use of plant-beneficial rhizobacteria may not only improve biocontrol and reduce the dependence on environmentally unfriendly pesticides but also increase plant growth and crop productivity. Among these strains, PGPB are considered to be keys for application in agriculture, enabling more sustainable agriculture. In this study, we explored the application of PGPB in the bioremediation of contaminated environments by a combination of PGPB and HM hyperaccumulator plants. To this end, the HM-resistant PGPB strain PGP15 was isolated and characterized. Similar to many other PGPB, strain PGP15 could significantly increase the growth of host plants in both the aerial and underground parts (Figure 3). Consistently, many PGPB-related genes were identified in the PGP15 genome, such as genes involved in cobalamin biosynthesis, acetolactate synthase, agmatinase, siderophore biosynthesis, and petrobactin biosynthesis. These genes and metabolic pathways have been reported as one of the important mechanisms utilized by certain PGPB with plant growth-promoting activity (Compant et al., 2005; Palacios et al., 2014; Kierul et al., 2015; Xu et al., 2018; Yi et al., 2018; Borker et al., 2021). More importantly, the PGP15 strain markedly improved Cd accumulation (Figure 3) in *S. nigrum* while alleviating Cd-induced stress in *S. nigrum* (Figure 3). Furthermore, *S. nigrum* has been reported to be a Cd hyperaccumulator plant (Sun et al., 2008; Chen et al., 2014). Our results showed that strain PGP15 could (1) promote the growth of *S. nigrum*, (2) increase Cd accumulation in *S. nigrum*, and (3) alleviate Cd-induced stress in *S. nigrum*. These results suggested that strain PGP15 could help to overcome the limits of phytoremediation alone, that is, small biomass, slow growth rate of plants, and HM toxicity to plants. The interactions between strain PGP15 and *S. nigrum* showed that the PGPB-hyperaccumulator plant collaborative pattern could have promising application potential for the bioremediation of Cd-contaminated soils.

Although many different soil bacteria have been isolated and considered to be PGPB (Compant et al., 2010; Glick, 2012; Xu et al., 2018; He et al., 2020; Chen et al., 2022), the mechanisms underlying plant promotion properties by HMT-PGPB in HM-contaminated soils are still largely unknown. With advances in sequencing technologies, the genomes of different strains could be obtained more quickly with lower cost, facilitating comparative genomic analysis. Comparing the genomes of PGPB and non-PGPB with close phylogenetic relationships could improve the understanding of the mechanism underlying the plant growth promotion process by PGPB. In this study, we compared the genome of strain PGP15 with the genomes of three closely related strains. We show that the PGP15 genome had high similarity with the other genomes, such as similar GC content, high ANI, and synteny (Figures 6A–C). In addition, more than 4,041 genes (71% of genes in the chromosome) were identified in LCBs (Figure 6D), and 5,121 (83.6% of total genes) genes of strain PGP15 were clustered into OGs with at least one other stain (Figure 7A), showing that these genes of strain PGP15 had orthologous genes in closely related species. Furthermore, most of the predicted genes associated with plant growth promotion identified in PGP15 have orthologous genes in the three other strains (Table 4). These phenomena suggest that the core genes that define the fundamental metabolic capabilities of a bacterium are not usually necessary for plant growth promotion (Huang et al., 2013). Similar results were also detected in other genera, showing that not all strains of a particular bacterial genus that have similar genetic makeup are PGPB. For example, some strains of *Pseudomonas* may actively promote plant growth, while other strains of the same genus have no measurable effect on plants (Mishra et al., 2017; Pires et al., 2017).

Bacteria are known for their ability to adapt to different environments, including extreme ones. For example, strain PGP15 showed high tolerance to Cd stresses (Figure 2). To date, many mechanisms have been suggested to explain the survival and tolerance that allow bacteria to live under different environmental conditions (Casacuberta and González, 2013). Among them, TEs have been reported as one of the fundamental forces supporting the variety of mechanisms of environmental adaptation (González et al., 2010; Schrader and Schmitz, 2019; Catlin and Josephs, 2022), as TEs are powerful mutagens that generate genomic variations. Consistently, although our comparative genomic analysis showed high similarity among the PGP15 genome and genomes of other closely related species, many PGP15-specific OGs and genes were detected. Interestingly, many of these PGP15-specific OGs are involved in TE and heavy metal binding and transportation. The results suggested that TEs might be related to the adaptive evolution of the PGP15 genome to achieve high heavy metal tolerance. Although mutations induced by TEs usually have deleterious effects, many studies have shown that TE-induced mutations are important for adaptation under environmental stresses in both prokaryotes and eukaryotes (Kidwell and Lisch, 2000; Casacuberta and González, 2013; Siguier et al., 2014; Rech et al., 2019). For example, TEs are reported to mediate metal resistance in the bacterium *Cupriavidus metallidurans* (Monchy et al., 2007; Mijnendonckx et al., 2011) and the fungus *Paecilomyces variotii* (Urquhart et al., 2022). TE-induced mutations can interrupt the promoter sequences of a gene and/or generate large genomic rearrangements involving several genes, which lead to an increase in host fitness (Ullastres et al., 2016). In addition, TEs could also increase genetic variation by horizontal gene transfer (HGT), one of the major forces in the evolution of the prokaryotic genome, which plays a crucial role in the processes of adaptive evolution for environmental stress resistance (Koonin et al., 2001; Thomas and Nielsen, 2005; Aminov, 2011; Sun et al., 2019; Rodríguez-Beltrán et al., 2021).

Different databases were used for genome annotation, which showed variations in the numbers of annotated genes. The difference in numbers of annotated genes might result from the capacity and focus of databases. In addition, many unannotated genes were detected, and many annotated genes were assigned to “hypothetical proteins,” suggesting that further functional studies are needed. In future studies, a deep analysis using transcriptomics, proteomics, and gene mutation could be highly useful in clarifying the complex interactions between strain PGP15 and *S. nigrum* under Cd stress, which will pave the way for the application of the PGPB-hyperaccumulator collaborative system in the future bioremediation of HM-contaminated environments.

## Data Availability Statement

The datasets presented in this study can be found in online repositories. The names of the repository/repositories and accession number(s) can be found at: NCBI—PRJNA824683, SAMN27411698.

## Author Contributions

CC and XX designed and led the overall study. YZ, SZ, SL, JP, and QZ carried out the experiments and measurements. YZ, HZ, and SZ analyzed the data. LZ, YC, and ZS provided expert advice. CC and XX wrote the draft manuscript. YZ, XX, and CC revised the manuscript. All authors read and approved the final manuscript.

## Funding

This work was supported by grants from the National Natural Science Foundation of China (41977120 and 31770404), the Key Research and Development Program of Jiangsu Province (BE2021718), the National Key Research and Development Program of China (2016YFD0800803), and the China Agriculture Research System (CARS-10-B24).

## Conflict of Interest

The authors declare that the research was conducted in the absence of any commercial or financial relationships that could be construed as a potential conflict of interest.

## Publisher’s Note

All claims expressed in this article are solely those of the authors and do not necessarily represent those of their affiliated organizations, or those of the publisher, the editors and the reviewers. Any product that may be evaluated in this article, or claim that may be made by its manufacturer, is not guaranteed or endorsed by the publisher.

## Footnotes

^1^ https://nanoporetech.com/community
