# Supplementary material for: Enhancing the Phytoremediation of Heavy Metals by Combining Hyperaccumulator and Heavy Metal-Resistant Plant Growth-Promoting Bacteria
Source: Front Plant Sci. 2022 Jun 2;13:912350. doi: 10.3389/fpls.2022.912350 (PMC9201774; doi:10.3389/fpls.2022.912350)
Supplement: Supplementary file 1 [file Data_Sheet_1.PDF]

Supplementary Material

1 Supplementary Tables

Table S1 Annotation of genes in the PGP15 genome.

Annotation database	No. of genes	Percentage (%)
Uniprot	3558	58.10
Pfam	5020	81.97
Refseq	5788	94.51
Nr	4266	69.66
Tigrfam	3059	49.95
GO	3268	53.36
KEGG	1395	22.78
COG	2204	35.99
Annotation	5834	95.26
All	6124	100

Table S2 Description of PGP15-specific ortholog groups (OGs).

OG_ID	Genes	Descriptions
OG0006	g_00633,g_01082,g_01998,g_	IS3 family transposase
OG0008	g_01522,g_01999,g_02481,g_	transposase
OG0011	g_00632,g_01081,g_02749,g_	-
OG0018	g_01361,g_02700,g_03263,g_	IS3 family transposase
OG0045	g_01521,g_02709,g_02995,g_	IS3 family transposase
OG0048	g_01362,g_03264,g_04612,g_	transposase
OG4012	g_01039,g_05877,g_05954	S-layer homology domain-containing protein
OG4023	g_05962,g_05963,g_05964	hypothetical protein
OG4024	g_06090,g_06091,g_06092	glycopeptide, sublancin family
OG4678	g_00842,g_04880	IS200/IS605 family element transposase accessory
OG4679	g_02193,g_02621	-
OG4680	g_02653,g_04352	N-acetylmuramoyl-L-alanine amidase

OG4681	g_02698,g_06065	serine hydrolase
OG4682	g_03202,g_05973	hypothetical protein
OG4683	g_03223,g_05893	1-deoxy-D-xylulose-5-phosphate reductoisomerase
OG4684	g_03224,g_05894	MFS transporter
OG4685	g_03337,g_06045	amino acid permease
OG4686	g_03475,g_06031	PLP-dependent aminotransferase family protein
OG4687	g_03611,g_05905	Transcriptional repressor
OG4688	g_04660,g_04661	hypothetical protein
OG4689	g_04857,g_04859	hypothetical protein
OG4690	g_05703,g_05709	IS200/IS605 family element transposase accessory
OG4691	g_05726,g_05728	MarR family transcriptional regulator
OG4692	g_05847,g_06112	IS4 family transposase
OG4712	g_00238,g_00241	hypothetical protein
OG4713	g_00964,g_00970	DUF4176 domain-containing protein
OG4714	g_00983,g_03399	SMI1/KNR4 family protein
OG4715	g_00998,g_04411	DUF4064 domain-containing protein
OG4716	g_02652,g_04353	hypothetical protein
OG4717	g_02699,g_02856	NUDIX domain-containing protein
OG4718	g_03183,g_06041	DUF4046 domain-containing protein
OG4719	g_03225,g_05895	ABC transporter substrate-binding protein
OG4720	g_03322,g_05705	hypothetical protein
OG4721	g_03388,g_03392	hypothetical protein
OG4722	g_03397,g_03401	IS110 family transposase
OG4723	g_03398,g_03402	recombinase family protein
OG4724	g_05898,g_05900	nucleotide pyrophosphohydrolase
OG4725	g_05917,g_05918	hypothetical protein
OG4726	g_05937,g_05939	hypothetical protein
OG4727	g_05979,g_05987	hypothetical protein
OG4728	g_06005,g_06085	hypothetical protein

Table S3 The unique genes identified in the PGP15 genome.

Genes	Source	Uniprot annotation

g_00234	Chromosome	-
g_00240	Chromosome	-
g_00246	Chromosome	-
g_00247	Chromosome	-
g_00458	Chromosome	-
g_00499	Chromosome	-
g_00534	Chromosome	-
g_00566	Chromosome	-
g_00576	Chromosome	-
g_00579	Chromosome	-
g_00609	Chromosome	-
g_00621	Chromosome	-
g_00650	Chromosome	-
g_00714	Chromosome	-
g_00815	Chromosome	-
g_00816	Chromosome	-
g_00912	Chromosome	-
g_00922	Chromosome	-
g_00923	Chromosome	-
g_00924	Chromosome	Protein translocase subunit SecA 2`
g_00927	Chromosome	-
g_00929	Chromosome	S-layer protein`
g_00940	Chromosome	ESAT-6 secretion system extracellular protein A`
g_00941	Chromosome	-
g_00944	Chromosome	-
g_00947	Chromosome	-
g_00949	Chromosome	-
g_00963	Chromosome	-
g_00968	Chromosome	Ribonuclease BC_0920`
g_00971	Chromosome	-
g_00973	Chromosome	-
g_00976	Chromosome	-
g_00977	Chromosome	-

g_00980	Chromosome	-
g_00982	Chromosome	-
g_00990	Chromosome	-
g_00994	Chromosome	-
g_00997	Chromosome	-
g_01007	Chromosome	-
g_01010	Chromosome	Probable 1-alkyl-2-acetylglycerophosphocholine esterase`
g_01011	Chromosome	-
g_01014	Chromosome	-
g_01018	Chromosome	-
g_01019	Chromosome	-
g_01091	Chromosome	-
g_01104	Chromosome	-
g_01109	Chromosome	-
g_01124	Chromosome	-
g_01132	Chromosome	-
g_01133	Chromosome	Uncharacterized HTH-type transcriptional regulator YobV`
g_01166	Chromosome	-
g_01169	Chromosome	-
g_01171	Chromosome	-
g_01197	Chromosome	-
g_01248	Chromosome	-
g_01250	Chromosome	-
g_01306	Chromosome	-
g_01360	Chromosome	-
g_01369	Chromosome	-
g_01373	Chromosome	-
g_01389	Chromosome	Ribonucleoside-diphosphate reductase subunit alpha`
g_01432	Chromosome	-
g_01498	Chromosome	-
g_01598	Chromosome	-
g_01613	Chromosome	Uncharacterized protein YcbJ`

g_01773	Chromosome	Metallo-beta-lactamase domain-containing protein 2`
g_01830	Chromosome	Uncharacterized protein YraL`
g_01833	Chromosome	-
g_01835	Chromosome	-
g_01836	Chromosome	NADPH-dependent reductive aminase`
g_01837	Chromosome	-
g_01839	Chromosome	-
g_01865	Chromosome	-
g_01875	Chromosome	-
g_01882	Chromosome	-
g_01889	Chromosome	-
g_01897	Chromosome	-
g_01898	Chromosome	Arsenical resistance operon repressor`
g_01899	Chromosome	Ferredoxin--NADP reductase`
g_01900	Chromosome	-
g_01901	Chromosome	Uncharacterized oxidoreductase CzcO-like`
g_01902	Chromosome	-
g_01903	Chromosome	HTH-type transcriptional repressor AseR`
g_01904	Chromosome	Arsenite resistance protein ArsB`
g_01905	Chromosome	Arsenate reductase 1`
g_01906	Chromosome	Arsenical resistance operon trans-acting repressor ArsD`
g_01907	Chromosome	Arsenical pump-driving ATPase`
g_01908	Chromosome	-
g_01910	Chromosome	-
g_01911	Chromosome	Uncharacterized membrane protein YmcC`
g_01925	Chromosome	-
g_01978	Chromosome	Alkaline phosphatase synthesis sensor protein PhoR`
g_02010	Chromosome	-
g_02011	Chromosome	-
g_02027	Chromosome	-
g_02028	Chromosome	-
g_02029	Chromosome	-
g_02030	Chromosome	-

g_02031	Chromosome	-
g_02032	Chromosome	-
g_02033	Chromosome	-
g_02034	Chromosome	Ribonuclease BC_0920`
g_02039	Chromosome	-
g_02097	Chromosome	-
g_02105	Chromosome	-
g_02106	Chromosome	-
g_02107	Chromosome	-
g_02108	Chromosome	-
g_02109	Chromosome	-
g_02110	Chromosome	-
g_02112	Chromosome	-
g_02113	Chromosome	-
g_02143	Chromosome	Antitoxin BC_0921`
g_02158	Chromosome	-
g_02165	Chromosome	-
g_02173	Chromosome	-
g_02174	Chromosome	-
g_02178	Chromosome	-
g_02183	Chromosome	-
g_02187	Chromosome	-
g_02188	Chromosome	-
g_02189	Chromosome	-
g_02190	Chromosome	-
g_02191	Chromosome	-
g_02196	Chromosome	Uncharacterized protein YrkC`
g_02197	Chromosome	-
g_02198	Chromosome	-
g_02199	Chromosome	-
g_02203	Chromosome	Uncharacterized protein HI_1410`
g_02212	Chromosome	-

g_02217	Chromosome	-
g_02232	Chromosome	-
g_02273	Chromosome	-
g_02274	Chromosome	Putative transposase in snaA-snaB intergenic region`
g_02275	Chromosome	-
g_02303	Chromosome	ADP-ribose pyrophosphatase`
g_02365	Chromosome	-
g_02371	Chromosome	-
g_02418	Chromosome	-
g_02427	Chromosome	-
g_02450	Chromosome	5-amino-6-(5-phospho-D-ribitylamino)uracil phosphatase YitU`
g_02452	Chromosome	-
g_02453	Chromosome	-
g_02457	Chromosome	-
g_02458	Chromosome	-
g_02459	Chromosome	-
g_02460	Chromosome	-
g_02461	Chromosome	-
g_02462	Chromosome	-
g_02480	Chromosome	Putative transposase InsK for insertion sequence element IS150`
g_02506	Chromosome	-
g_02507	Chromosome	-
g_02518	Chromosome	-
g_02543	Chromosome	-
g_02553	Chromosome	Uncharacterized protein YnaE`
g_02570	Chromosome	Transposon Tn1546 resolvase`
g_02573	Chromosome	-
g_02574	Chromosome	-
g_02582	Chromosome	-
g_02584	Chromosome	-
g_02585	Chromosome	Uncharacterized membrane protein YkoS`
g_02587	Chromosome	-
g_02592	Chromosome	-

g_02598	Chromosome	Cell wall hydrolase CwlJ`
g_02605	Chromosome	-
g_02606	Chromosome	-
g_02619	Chromosome	-
g_02620	Chromosome	-
g_02622	Chromosome	-
g_02623	Chromosome	Probable integrase/recombinase YoeC`
g_02626	Chromosome	-
g_02628	Chromosome	-
g_02629	Chromosome	-
g_02630	Chromosome	-
g_02631	Chromosome	-
g_02632	Chromosome	-
g_02633	Chromosome	-
g_02634	Chromosome	-
g_02635	Chromosome	-
g_02636	Chromosome	-
g_02637	Chromosome	-
g_02642	Chromosome	-
g_02645	Chromosome	-
g_02646	Chromosome	-
g_02649	Chromosome	-
g_02651	Chromosome	-
g_02654	Chromosome	-
g_02655	Chromosome	-
g_02656	Chromosome	-
g_02657	Chromosome	-
g_02658	Chromosome	-
g_02672	Chromosome	Cytochrome P450(BM-1)`
g_02696	Chromosome	-
g_02697	Chromosome	-
g_02701	Chromosome	-

g_02702	Chromosome	-
g_02735	Chromosome	-
g_02740	Chromosome	-
g_02748	Chromosome	-
g_02751	Chromosome	-
g_02774	Chromosome	Transposase A from transposon Tn554`
g_02775	Chromosome	Tyrosine recombinase XerC`
g_02776	Chromosome	-
g_02778	Chromosome	-
g_02779	Chromosome	-
g_02780	Chromosome	-
g_02785	Chromosome	-
g_02794	Chromosome	-
g_02795	Chromosome	Bacitracin synthase 1`
g_02796	Chromosome	Tryptophan decarboxylase`
g_02798	Chromosome	-
g_02799	Chromosome	-
g_02801	Chromosome	-
g_02824	Chromosome	-
g_02826	Chromosome	-
g_02830	Chromosome	Uncharacterized phosphatase PhoE`
g_02831	Chromosome	-
g_02839	Chromosome	-
g_02852	Chromosome	-
g_02880	Chromosome	-
g_02903	Chromosome	-
g_02929	Chromosome	-
g_02937	Chromosome	-
g_02939	Chromosome	-
g_02952	Chromosome	-
g_02966	Chromosome	-
g_02971	Chromosome	-
g_02972	Chromosome	-

g_02973	Chromosome	-
g_02987	Chromosome	Bifunctional AAC/APH`
g_03024	Chromosome	Maltodextrin import ATP-binding protein MsmX`
g_03025	Chromosome	-
g_03026	Chromosome	-
g_03027	Chromosome	-
g_03030	Chromosome	Uncharacterized HTH-type transcriptional regulator YulB`
g_03062	Chromosome	-
g_03063	Chromosome	-
g_03068	Chromosome	Antitoxin YokJ`
g_03071	Chromosome	Probable RNA 2'-phosphotransferase`
g_03072	Chromosome	Probable RNA 2'-phosphotransferase`
g_03073	Chromosome	-
g_03074	Chromosome	-
g_03075	Chromosome	-
g_03076	Chromosome	-
g_03077	Chromosome	Ribonuclease BC_0920`
g_03100	Chromosome	Sensor histidine kinase YbdK`
g_03101	Chromosome	Uncharacterized transcriptional regulatory protein YbdJ`
g_03130	Chromosome	-
g_03139	Chromosome	-
g_03149	Chromosome	-
g_03150	Chromosome	-
g_03151	Chromosome	Spore coat protein F`
g_03152	Chromosome	-
g_03153	Chromosome	Uncharacterized protein YqcI`
g_03154	Chromosome	Putative amino acid efflux protein YcgF`
g_03155	Chromosome	Uncharacterized transporter YcgH`
g_03156	Chromosome	-
g_03157	Chromosome	Small, acid-soluble spore protein 1`
g_03162	Chromosome	-
g_03163	Chromosome	-

g_03179	Chromosome	Branched-chain amino acid transport system carrier protein BrnQ`
g_03181	Chromosome	Uncharacterized protein YdgD`
g_03182	Chromosome	Probable NAD-dependent malic enzyme 4`
g_03206	Chromosome	-
g_03228	Chromosome	Bacitracin export ATP-binding protein BceA`
g_03229	Chromosome	Bacitracin export permease protein BceB`
g_03230	Chromosome	-
g_03231	Chromosome	-
g_03241	Chromosome	Arsenical resistance operon repressor`
g_03242	Chromosome	-
g_03244	Chromosome	-
g_03266	Chromosome	-
g_03267	Chromosome	Bifunctional transcriptional activator/DNA repair enzyme AdaA`
g_03268	Chromosome	-
g_03270	Chromosome	Transposase for insertion sequence element IS231F`
g_03280	Chromosome	-
g_03285	Chromosome	-
g_03294	Chromosome	Probable sulfoacetate transporter SauU`
g_03295	Chromosome	Lactate utilization protein C`
g_03296	Chromosome	Lactate utilization protein B`
g_03298	Chromosome	HTH-type transcriptional regulator BsdA`
g_03300	Chromosome	-
g_03309	Chromosome	Bacillolysin`
g_03310	Chromosome	D-alanyl-D-alanine carboxypeptidase`
g_03320	Chromosome	-
g_03326	Chromosome	-
g_03331	Chromosome	Probable protein adenylyltransferase HI_0977`
g_03332	Chromosome	Diaminobutyrate--2-oxoglutarate transaminase`
g_03333	Chromosome	Carbamoyltransferase HypF2`
g_03334	Chromosome	Hydrogenase expression/formation protein HupE`
g_03335	Chromosome	Peroxyureidoacrylate/ureidoacrylate amidohydrolase RutB`
g_03336	Chromosome	Uncharacterized HTH-type transcriptional regulator YezC`
g_03338	Chromosome	-

g_03339	Chromosome	-
g_03340	Chromosome	-
g_03351	Chromosome	-
g_03358	Chromosome	-
g_03363	Chromosome	-
g_03368	Chromosome	-
g_03369	Chromosome	-
g_03370	Chromosome	-
g_03373	Chromosome	-
g_03377	Chromosome	-
g_03384	Chromosome	-
g_03385	Chromosome	-
g_03390	Chromosome	-
g_03391	Chromosome	-
g_03395	Chromosome	-
g_03396	Chromosome	-
g_03400	Chromosome	-
g_03422	Chromosome	Putative transposase in snaA-snaB intergenic region`
g_03423	Chromosome	-
g_03424	Chromosome	Uncharacterized protein YnaC`
g_03425	Chromosome	-
g_03427	Chromosome	-
g_03449	Chromosome	-
g_03457	Chromosome	-
g_03466	Chromosome	-
g_03467	Chromosome	-
g_03476	Chromosome	Putative glutamine amidotransferase-like protein YfeJ`
g_03477	Chromosome	-
g_03486	Chromosome	-
g_03487	Chromosome	-
g_03514	Chromosome	-
g_03526	Chromosome	Uncharacterized aromatic compound monooxygenase YhjG`

g_03576	Chromosome	-
g_03590	Chromosome	-
g_03596	Chromosome	-
g_03597	Chromosome	Response regulator aspartate phosphatase K`
g_03608	Chromosome	-
g_03609	Chromosome	Alpha-hemolysin`
g_03610	Chromosome	Uncharacterized cell wall amidase pXO2-
g_03751	Chromosome	Putative transposase InsQ for insertion sequence element IS609`
g_03787	Chromosome	-
g_03788	Chromosome	-
g_03789	Chromosome	-
g_03790	Chromosome	LexA repressor`
g_03799	Chromosome	-
g_03811	Chromosome	-
g_03845	Chromosome	-
g_03853	Chromosome	-
g_03895	Chromosome	-
g_04091	Chromosome	-
g_04105	Chromosome	Putative transposase in snaA-snaB intergenic region`
g_04134	Chromosome	-
g_04244	Chromosome	-
g_04256	Chromosome	-
g_04282	Chromosome	-
g_04342	Chromosome	-
g_04350	Chromosome	-
g_04351	Chromosome	-
g_04354	Chromosome	-
g_04355	Chromosome	-
g_04356	Chromosome	-
g_04357	Chromosome	-
g_04358	Chromosome	-
g_04359	Chromosome	-
g_04360	Chromosome	-

g_04362	Chromosome	-
g_04377	Chromosome	-
g_04379	Chromosome	-
g_04384	Chromosome	-
g_04385	Chromosome	-
g_04386	Chromosome	-
g_04387	Chromosome	-
g_04388	Chromosome	-
g_04389	Chromosome	-
g_04390	Chromosome	-
g_04391	Chromosome	-
g_04392	Chromosome	-
g_04393	Chromosome	-
g_04395	Chromosome	-
g_04396	Chromosome	Transition state regulatory protein AbrB`
g_04399	Chromosome	-
g_04404	Chromosome	-
g_04405	Chromosome	-
g_04406	Chromosome	-
g_04412	Chromosome	-
g_04414	Chromosome	-
g_04570	Chromosome	-
g_04581	Chromosome	-
g_04610	Chromosome	-
g_04656	Chromosome	-
g_04664	Chromosome	-
g_04665	Chromosome	-
g_04666	Chromosome	-
g_04667	Chromosome	-
g_04668	Chromosome	-
g_04669	Chromosome	-
g_04670	Chromosome	-

g_04671	Chromosome	-
g_04672	Chromosome	-
g_04673	Chromosome	-
g_04675	Chromosome	-
g_04676	Chromosome	-
g_04739	Chromosome	-
g_04756	Chromosome	-
g_04785	Chromosome	-
g_04831	Chromosome	-
g_04858	Chromosome	-
g_04933	Chromosome	-
g_04934	Chromosome	-
g_04945	Chromosome	-
g_04955	Chromosome	Uncharacterized 39.9 kDa protein in amylase 3'region`
g_04995	Chromosome	-
g_04996	Chromosome	Macrolide export ATP-binding/permease protein MacB`
g_04998	Chromosome	-
g_05004	Chromosome	-
g_05033	Chromosome	Bacitracin export permease protein BceB`
g_05034	Chromosome	Bacitracin export ATP-binding protein BceA`
g_05036	Chromosome	-
g_05037	Chromosome	-
g_05038	Chromosome	-
g_05046	Chromosome	-
g_05047	Chromosome	ECF RNA polymerase sigma factor SigW`
g_05076	Chromosome	-
g_05083	Chromosome	-
g_05215	Chromosome	-
g_05216	Chromosome	-
g_05234	Chromosome	-
g_05240	Chromosome	-
g_05244	Chromosome	-
g_05251	Chromosome	Methionine--tRNA ligase 1`

g_05265	Chromosome	-
g_05273	Chromosome	-
g_05300	Chromosome	-
g_05326	Chromosome	Uncharacterized protein YxeA`
g_05368	Chromosome	-
g_05402	Chromosome	-
g_05403	Chromosome	-
g_05429	Chromosome	-
g_05459	Chromosome	-
g_05461	Chromosome	Teichoic acids export ATP-binding protein TagH`
g_05462	Chromosome	Teichoic acid translocation permease protein TagG`
g_05463	Chromosome	UDP-N-acetyl-D-mannosamine dehydrogenase`
g_05464	Chromosome	-
g_05465	Chromosome	-
g_05466	Chromosome	Poly(ribitol-phosphate) beta-glucosyltransferase`
g_05467	Chromosome	Glycosyltransferase GlyA`
g_05468	Chromosome	-
g_05469	Chromosome	Uncharacterized glycosyltransferase MJ1069`
g_05472	Chromosome	Uncharacterized protein YadE`
g_05514	Chromosome	-
g_05515	Chromosome	-
g_05517	Chromosome	-
g_05554	Chromosome	-
g_05584	Chromosome	-
g_05621	Chromosome	-
g_05642	Chromosome	-
g_05644	Chromosome	-
g_05682	Plasmid 1	Zinc-binding lipoprotein AdcA`
g_05683	Plasmid 1	Transposase for insertion sequence element IS231C`
g_05684	Plasmid 1	-
g_05685	Plasmid 1	Probable isoprenylcysteine alpha-carbonyl methylesterase ICMEL2`
g_05686	Plasmid 1	Probable sugar efflux transporter`

g_05688	Plasmid 1	-
g_05691	Plasmid 1	-
g_05692	Plasmid 1	-
g_05701	Plasmid 1	-
g_05710	Plasmid 1	RNA-binding protein Hfq`
g_05711	Plasmid 1	Transcriptional repressor PagR`
g_05712	Plasmid 1	Probable disulfide formation protein C 2`
g_05713	Plasmid 1	-
g_05715	Plasmid 1	Linear gramicidin dehydrogenase LgrE`
g_05716	Plasmid 1	ABC transporter F family member 5`
g_05719	Plasmid 1	ATP-binding protein SyrD`
g_05720	Plasmid 1	Granaticin polyketide putative beta-ketoacyl synthase 1`
g_05721	Plasmid 1	-
g_05722	Plasmid 1	Long-chain-fatty-acid--CoA ligase`
g_05723	Plasmid 1	Probable acetolactate synthase large subunit`
g_05724	Plasmid 1	Aldehyde dehydrogenase 1`
g_05725	Plasmid 1	-
g_05727	Plasmid 1	Protein FmtA`
g_05729	Plasmid 1	-
g_05731	Plasmid 1	-
g_05732	Plasmid 1	Uncharacterized protein pXO1-01/BXA0003/GBAA_pXO1_0003`
g_05733	Plasmid 1	-
g_05734	Plasmid 1	-
g_05735	Plasmid 1	-
g_05736	Plasmid 1	-
g_05737	Plasmid 1	-
g_05738	Plasmid 1	-
g_05739	Plasmid 1	Ribonuclease BC_0920`
g_05741	Plasmid 1	-
g_05742	Plasmid 1	S-layer protein EA1`
g_05744	Plasmid 1	-
g_05745	Plasmid 1	-
g_05750	Plasmid 1	Response regulator aspartate phosphatase A`

g_05751	Plasmid 1	-
g_05753	Plasmid 1	-
g_05754	Plasmid 1	-
g_05755	Plasmid 1	-
g_05765	Plasmid 1	-
g_05766	Plasmid 1	-
g_05767	Plasmid 1	-
g_05768	Plasmid 1	-
g_05769	Plasmid 1	-
g_05770	Plasmid 1	-
g_05771	Plasmid 1	-
g_05772	Plasmid 1	-
g_05773	Plasmid 1	-
g_05774	Plasmid 1	-
g_05775	Plasmid 1	Uncharacterized protein in xynA 3'region (Fragment)`
g_05776	Plasmid 1	-
g_05777	Plasmid 1	-
g_05778	Plasmid 1	-
g_05779	Plasmid 1	-
g_05780	Plasmid 1	-
g_05786	Plasmid 1	Putative glycerophosphodiester phosphodiesterase YhdW`
g_05787	Plasmid 1	dTDP-4-amino-4,6-dideoxy-D-glucose acyltransferase`
g_05788	Plasmid 1	Protein Mb3436c`
g_05791	Plasmid 1	-
g_05792	Plasmid 1	Inner membrane protein YjdF`
g_05793	Plasmid 1	Undecaprenyl-diphosphatase BcrC`
g_05794	Plasmid 1	-
g_05795	Plasmid 1	Small, acid-soluble spore protein H`
g_05796	Plasmid 1	UPF0702 transmembrane protein YdfS`
g_05797	Plasmid 1	-
g_05798	Plasmid 1	-
g_05800	Plasmid 1	RNA-directed DNA polymerase from retron EC86`

g_05801	Plasmid 1	-
g_05802	Plasmid 1	-
g_05803	Plasmid 1	-
g_05804	Plasmid 1	-
g_05806	Plasmid 1	-
g_05813	Plasmid 1	Uncharacterized protein YwoF`
g_05814	Plasmid 1	-
g_05815	Plasmid 1	-
g_05816	Plasmid 1	-
g_05817	Plasmid 1	-
g_05818	Plasmid 1	-
g_05819	Plasmid 1	-
g_05822	Plasmid 1	-
g_05823	Plasmid 1	-
g_05824	Plasmid 1	-
g_05825	Plasmid 1	-
g_05826	Plasmid 1	-
g_05830	Plasmid 1	-
g_05832	Plasmid 1	-
g_05833	Plasmid 1	-
g_05834	Plasmid 1	Ubiquinone/menaquinone biosynthesis C-methyltransferase UbiE`
g_05838	Plasmid 1	Homocysteine S-methyltransferase YbgG`
g_05839	Plasmid 1	Uncharacterized amino acid permease YbgF`
g_05841	Plasmid 1	Transposase for insertion sequence element IS231F`
g_05855	Plasmid 1	-
g_05857	Plasmid 1	-
g_05858	Plasmid 1	-
g_05859	Plasmid 1	-
g_05860	Plasmid 1	-
g_05861	Plasmid 1	-
g_05862	Plasmid 1	DNA translocase SpoIIIE`
g_05863	Plasmid 1	-
g_05864	Plasmid 1	-

g_05865	Plasmid 1	-
g_05866	Plasmid 1	-
g_05867	Plasmid 1	-
g_05868	Plasmid 1	-
g_05870	Plasmid 1	Uncharacterized mitochondrial protein ymf40`
g_05872	Plasmid 1	-
g_05874	Plasmid 1	-
g_05878	Plasmid 1	Intracellular alkaline protease`
g_05879	Plasmid 1	Aminopeptidase YwaD`
g_05880	Plasmid 1	-
g_05881	Plasmid 1	Ulilysin`
g_05882	Plasmid 1	-
g_05884	Plasmid 1	-
g_05887	Plasmid 1	-
g_05889	Plasmid 1	-
g_05890	Plasmid 1	-
g_05896	Plasmid 1	-
g_05897	Plasmid 1	-
g_05899	Plasmid 1	-
g_05904	Plasmid 1	-
g_05908	Plasmid 1	Probable methyl-accepting chemotaxis protein BT9727_0355`
g_05910	Plasmid 1	-
g_05913	Plasmid 1	-
g_05914	Plasmid 1	-
g_05915	Plasmid 1	UPF0276 protein AM1_3026`
g_05916	Plasmid 1	-
g_05919	Plasmid 1	-
g_05920	Plasmid 1	Lipid A export ATP-binding/permease protein MsbA`
g_05922	Plasmid 1	Uncharacterized protein YlbO`
g_05923	Plasmid 1	-
g_05926	Plasmid 1	-
g_05927	Plasmid 1	-

g_05928	Plasmid 1	-
g_05929	Plasmid 1	-
g_05930	Plasmid 1	-
g_05931	Plasmid 1	-
g_05932	Plasmid 1	-
g_05934	Plasmid 1	-
g_05935	Plasmid 1	Na(+), Li(+), K(+)/H(+) antiporter`
g_05936	Plasmid 1	-
g_05938	Plasmid 1	-
g_05940	Plasmid 1	-
g_05941	Plasmid 1	-
g_05942	Plasmid 1	-
g_05943	Plasmid 1	-
g_05944	Plasmid 1	DNA translocase FtsK`
g_05945	Plasmid 1	-
g_05946	Plasmid 1	-
g_05950	Plasmid 1	-
g_05951	Plasmid 1	-
g_05952	Plasmid 1	-
g_05953	Plasmid 1	-
g_05955	Plasmid 1	-
g_05956	Plasmid 1	-
g_05957	Plasmid 1	-
g_05958	Plasmid 1	-
g_05959	Plasmid 1	-
g_05960	Plasmid 1	-
g_05961	Plasmid 1	-
g_05965	Plasmid 1	-
g_05966	Plasmid 1	-
g_05967	Plasmid 1	-
g_05968	Plasmid 1	Transposase for insertion sequence element IS231E`
g_05969	Plasmid 1	Transposase for insertion sequence element IS231A`
g_05972	Plasmid 1	-

g_05974	Plasmid 1	-
g_05978	Plasmid 1	-
g_05981	Plasmid 1	-
g_05985	Plasmid 1	-
g_05986	Plasmid 1	-
g_05988	Plasmid 1	-
g_05989	Plasmid 1	-
g_05990	Plasmid 1	-
g_05991	Plasmid 1	-
g_05992	Plasmid 1	-
g_05993	Plasmid 1	-
g_05994	Plasmid 1	-
g_05996	Plasmid 1	Protein YkfC`
g_05998	Plasmid 1	-
g_05999	Plasmid 1	-
g_06000	Plasmid 1	-
g_06001	Plasmid 1	-
g_06002	Plasmid 1	Uncharacterized protein YrkO`
g_06003	Plasmid 1	-
g_06004	Plasmid 1	Foldase protein PrsA`
g_06006	Plasmid 1	-
g_06007	Plasmid 1	-
g_06008	Plasmid 1	-
g_06009	Plasmid 1	NLP effector protein 3`
g_06010	Plasmid 1	-
g_06019	Plasmid 1	-
g_06024	Plasmid 1	-
g_06025	Plasmid 1	-
g_06026	Plasmid 1	-
g_06027	Plasmid 1	-
g_06028	Plasmid 1	-
g_06029	Plasmid 1	-

g_06032	Plasmid 1	-
g_06035	Plasmid 1	-
g_06037	Plasmid 1	-
g_06039	Plasmid 1	Peptidoglycan-N-acetylglucosamine deacetylase BC_1974`
g_06043	Plasmid 1	-
g_06046	Plasmid 1	Uncharacterized protein in bps2 5'region (Fragment)`
g_06047	Plasmid 1	Phospholipase C`
g_06049	Plasmid 1	Putative peptidoglycan O-acetyltransferase YrhL`
g_06055	Plasmid 1	Transposase A from transposon Tn554`
g_06056	Plasmid 1	Transposase A from transposon Tn554`
g_06057	Plasmid 1	Tyrosine recombinase XerC`
g_06058	Plasmid 1	-
g_06059	Plasmid 1	Uncharacterized HTH-type transcriptional regulator AF_1627`
g_06060	Plasmid 1	-
g_06061	Plasmid 1	Spermine/spermidine acetyltransferase`
g_06062	Plasmid 1	ABC transporter permease protein YxdM`
g_06063	Plasmid 1	ABC transporter ATP-binding protein YxdL`
g_06067	Plasmid 1	-
g_06069	Plasmid 1	Violacein synthase`
g_06070	Plasmid 1	Transposons Tn1721 resolvase`
g_06073	Plasmid 1	Uncharacterized protein YvpB`
g_06077	Plasmid 1	-
g_06078	Plasmid 1	D-alanyl carrier protein`
g_06080	Plasmid 1	HTH-type transcriptional regulator LmrA`
g_06082	Plasmid 1	-
g_06083	Plasmid 1	-
g_06084	Plasmid 1	-
g_06086	Plasmid 1	-
g_06087	Plasmid 1	Lipid II flippase Amj`
g_06088	Plasmid 1	-
g_06089	Plasmid 1	-
g_06093	Plasmid 1	SPbeta prophage-derived sublancin-168-processing and transport ATP- [illegible]
g_06094	Plasmid 1	SPbeta prophage-derived disulfide bond formation protein A`

g_06095	Plasmid 1	SPbeta prophage-derived disulfide bond formation protein B`
g_06097	Plasmid 1	Virginiamycin B lyase`
g_06098	Plasmid 1	Uncharacterized protein YqcK`
g_06099	Plasmid 1	-
g_06102	Plasmid 1	-
g_06103	Plasmid 1	Transcriptional repressor SdpR`
g_06108	Plasmid 1	-
g_06109	Plasmid 1	Protein YkfC`
g_06113	Plasmid 1	Methylthioribose transporter`
g_06114	Plasmid 1	-
g_06115	Plasmid 1	-
g_06116	Plasmid 1	Uncharacterized protein YrhO`
g_06117	Plasmid 2	-
g_06118	Plasmid 2	-
g_06119	Plasmid 2	-
g_06120	Plasmid 2	-
g_06121	Plasmid 2	-
g_06122	Plasmid 2	-
g_06123	Plasmid 2	Uncharacterized 23.6 kDa protein`
g_06124	Plasmid 2	-
